# Supplementary material for: Selective sorting of ancestral introgression in maize and teosinte along an elevational cline
Source: PLoS Genet. 2021 Oct 11;17(10):e1009810. doi: 10.1371/journal.pgen.1009810 (PMC8530355; doi:10.1371/journal.pgen.1009810)
Supplement: S3 Table — (PDF) [file pgen.1009810.s003.pdf]

S3 Table. Spearman's rank correlation between genomewide admixture proportions (NGSAdmix) and recombination rate (or coding bp per cM) quintiles

group	feature	ancestry	Spearman's ρ	2.5%	97.5%
sympatric mexicana	recombination rate (cM/Mb)	maize	-1.00	-1.00	-0.90
sympatric mexicana	recombination rate (cM/Mb)	mexicana	1.00	0.80	1.00
sympatric mexicana	recombination rate (cM/Mb)	parviglumis	0.00	-0.90	0.90
sympatric maize	recombination rate (cM/Mb)	maize	-1.00	-1.00	-0.85
sympatric maize	recombination rate (cM/Mb)	mexicana	1.00	0.80	1.00
sympatric maize	recombination rate (cM/Mb)	parviglumis	0.70	0.10	0.95
reference parviglumis	recombination rate (cM/Mb)	maize	-0.50	-1.00	-0.35
reference parviglumis	recombination rate (cM/Mb)	mexicana	0.10	-0.95	0.50
reference parviglumis	recombination rate (cM/Mb)	parviglumis	0.50	0.30	1.00
reference mexicana	recombination rate (cM/Mb)	maize	-0.70	-1.00	-0.40
reference mexicana	recombination rate (cM/Mb)	mexicana	0.50	-0.30	0.80
reference mexicana	recombination rate (cM/Mb)	parviglumis	-0.10	-0.70	0.50
reference maize	recombination rate (cM/Mb)	maize	0.30	-0.25	0.70
reference maize	recombination rate (cM/Mb)	mexicana	0.00	-0.70	1.00
reference maize	recombination rate (cM/Mb)	parviglumis	-0.30	-0.70	0.30
sympatric mexicana	gene density (coding bp/cM)	maize	1.00	0.85	1.00
sympatric mexicana	gene density (coding bp/cM)	mexicana	-1.00	-1.00	-0.80
sympatric mexicana	gene density (coding bp/cM)	parviglumis	-0.70	-1.00	0.90
sympatric maize	gene density (coding bp/cM)	maize	1.00	0.90	1.00
sympatric maize	gene density (coding bp/cM)	mexicana	-1.00	-1.00	-0.90
sympatric maize	gene density (coding bp/cM)	parviglumis	-0.70	-0.90	0.15
reference parviglumis	gene density (coding bp/cM)	maize	0.30	0.00	0.90
reference parviglumis	gene density (coding bp/cM)	mexicana	0.70	-0.30	1.00
reference parviglumis	gene density (coding bp/cM)	parviglumis	-0.60	-0.90	-0.05
reference mexicana	gene density (coding bp/cM)	maize	0.90	0.30	0.95
reference mexicana	gene density (coding bp/cM)	mexicana	-0.40	-0.90	0.35
reference mexicana	gene density (coding bp/cM)	parviglumis	0.30	-0.60	0.90
reference maize	gene density (coding bp/cM)	maize	-0.90	-1.00	-0.10
reference maize	gene density (coding bp/cM)	mexicana	0.10	-0.80	0.80
reference maize	gene density (coding bp/cM)	parviglumis	0.90	0.10	0.90
