# Supplementary material for: Selective sorting of ancestral introgression in maize and teosinte along an elevational cline
Source: PLoS Genet. 2021 Oct 11;17(10):e1009810. doi: 10.1371/journal.pgen.1009810 (PMC8530355; doi:10.1371/journal.pgen.1009810)
Supplement: S4 Table — Best-fitting linear models for ancestry proportion predicted by an elevation by recombination rate interaction: mexicana ancestry ∼ elevation + r + elevation*r. Here, r is the recombination rate quintile, treated as numeric [0-4]. This model only uses ancestry estimates for sympatric individuals and is fit separately for maize and mexicana samples. (PDF) [file pgen.1009810.s004.pdf]

S4 Table. ***Mexicana* ancestry by elevation and recombination rate quintile.** Best-fitting linear models for ancestry proportion predicted by an elevation by recombination rate interaction: *mexicana* ancestry $\sim$ elevation + r + elevation*r. Here, r is the recombination rate quintile, treated as numeric [0-4]. This model only uses ancestry estimates for sympatric individuals and is fit separately for maize and *mexicana* samples.

group	term	estimate	std.error	statistic	p.value
sympatric maize	intercept	-0.147	0.028	-5.307	1.55E-07
sympatric maize	elevation (km)	0.099	0.013	7.630	8.78E-14
sympatric maize	r quintile	-0.114	0.011	-10.034	4.54E-22
sympatric maize	elevation*r quintile	0.077	0.005	14.484	3.27E-41
sympatric mexicana	intercept	0.045	0.034	1.325	1.86E-01
sympatric mexicana	elevation (km)	0.366	0.016	23.252	7.62E-94
sympatric mexicana	r quintile	0.086	0.014	6.242	6.67E-10
sympatric mexicana	elevation*r quintile	-0.031	0.006	-4.832	1.59E-06
