# Supplementary material for: Selective sorting of ancestral introgression in maize and teosinte along an elevational cline
Source: PLoS Genet. 2021 Oct 11;17(10):e1009810. doi: 10.1371/journal.pgen.1009810 (PMC8530355; doi:10.1371/journal.pgen.1009810)
Supplement: S5 Table — Confidence intervals are constructed using the percentile method and 10,000 bootstrap replicates created by randomly re-sampling 1 cM windows within quintiles. (PDF) [file pgen.1009810.s005.pdf]

S5 Table. Spearman's rank correlation between mean local ancestry and recombination rate (or coding bp per cM) at 1 cM genomic window resolution. Confidence intervals are constructed using the percentile method and 10,000 bootstrap replicates created by randomly re-sampling 1 cM windows within quintiles.

group	ancestry	feature	Spearman's ρ	2.5%	97.5%
symp. mexicana	maize	recombination rate (cM/Mb)	0.385	0.341	0.428
symp. mexicana	mexicana	recombination rate (cM/Mb)	-0.579	-0.610	-0.545
symp. mexicana	parviglumis	recombination rate (cM/Mb)	0.507	0.469	0.543
symp. maize	maize	recombination rate (cM/Mb)	-0.066	-0.117	-0.014
symp. maize	mexicana	recombination rate (cM/Mb)	0.011	-0.038	0.061
symp. maize	parviglumis	recombination rate (cM/Mb)	0.105	0.055	0.157
symp. mexicana	maize	gene density (coding bp/cM)	-0.262	-0.309	-0.212
symp. mexicana	mexicana	gene density (coding bp/cM)	0.423	0.381	0.464
symp. mexicana	parviglumis	gene density (coding bp/cM)	-0.385	-0.428	-0.341
symp. maize	maize	gene density (coding bp/cM)	0.050	-0.003	0.101
symp. maize	mexicana	gene density (coding bp/cM)	0.030	-0.022	0.080
symp. maize	parviglumis	gene density (coding bp/cM)	-0.099	-0.151	-0.048
