# Supplementary material for: Selective sorting of ancestral introgression in maize and teosinte along an elevational cline
Source: PLoS Genet. 2021 Oct 11;17(10):e1009810. doi: 10.1371/journal.pgen.1009810 (PMC8530355; doi:10.1371/journal.pgen.1009810)
Supplement: S6 Table — Pairwise diversity (π) and Watterson’s theta (θW) for samples clustering by PCA with the maize-allele at the inversion (PC1 > 0.2) or mexicana-allele at the inversion (PC1 < 0.4). Individuals heterozygous for the inversion (or ambiguous in PCA clustering) were excluded. Diversity estimates within the putative inversion region were calculated for each group using the same ANGSD/realSFS pipeline as genomewide diversity estimates. Only subspecies with > 5 samples in a cluster were analysed. (PDF) [file pgen.1009810.s006.pdf]

S6 Table. Diversity within maize and *mexicana* alleles at *inv9f*. Pairwise diversity (π) and Watterson's theta (θ_W) for samples clustering by PCA with the maize-allele at the inversion (PC1 > 0.2) or *mexicana*-allele at the inversion (PC1 < 0.4). Individuals heterozygous for the inversion (or ambiguous in PCA clustering) were excluded. Diversity estimates within the putative inversion region were calculated for each group using the same ANGSD/realSFS pipeline as genomewide diversity estimates. Only subspecies with > 5 samples in a cluster were analysed.

sample	π	θ_W
mexicana within mexicana inversion cluster	0.024	0.083
maize within mexicana inversion cluster	0.019	0.053
parviglumis within maize inversion cluster	0.013	0.016
maize within maize inversion cluster	0.011	0.022
