# Supplementary figures and images for: Selective sorting of ancestral introgression in maize and teosinte along an elevational cline
Source: PLoS Genet. 2021 Oct 11;17(10):e1009810. doi: 10.1371/journal.pgen.1009810 (PMC8530355; doi:10.1371/journal.pgen.1009810)

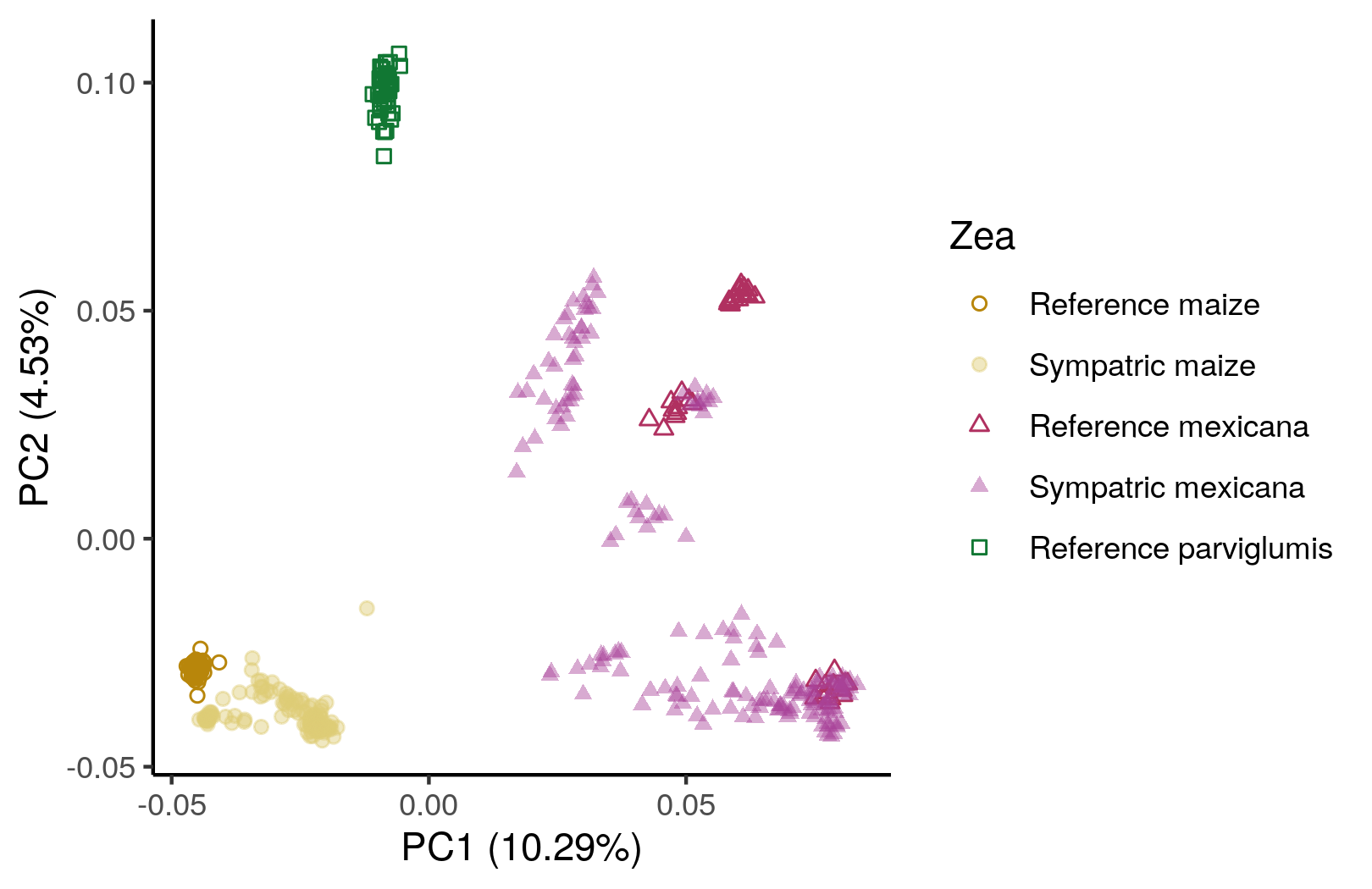

Supplement: S1 Fig — First and second principal components from the genomewide genetic covariance matrix relating all maize, mexicana and parviglumis individuals. PC1 aligns with a maize to mexicana ancestry gradient while PC2 separates out parviglumis ancestry from the other two Zea subspecies. (TIF) [file pgen.1009810.s008.tif]

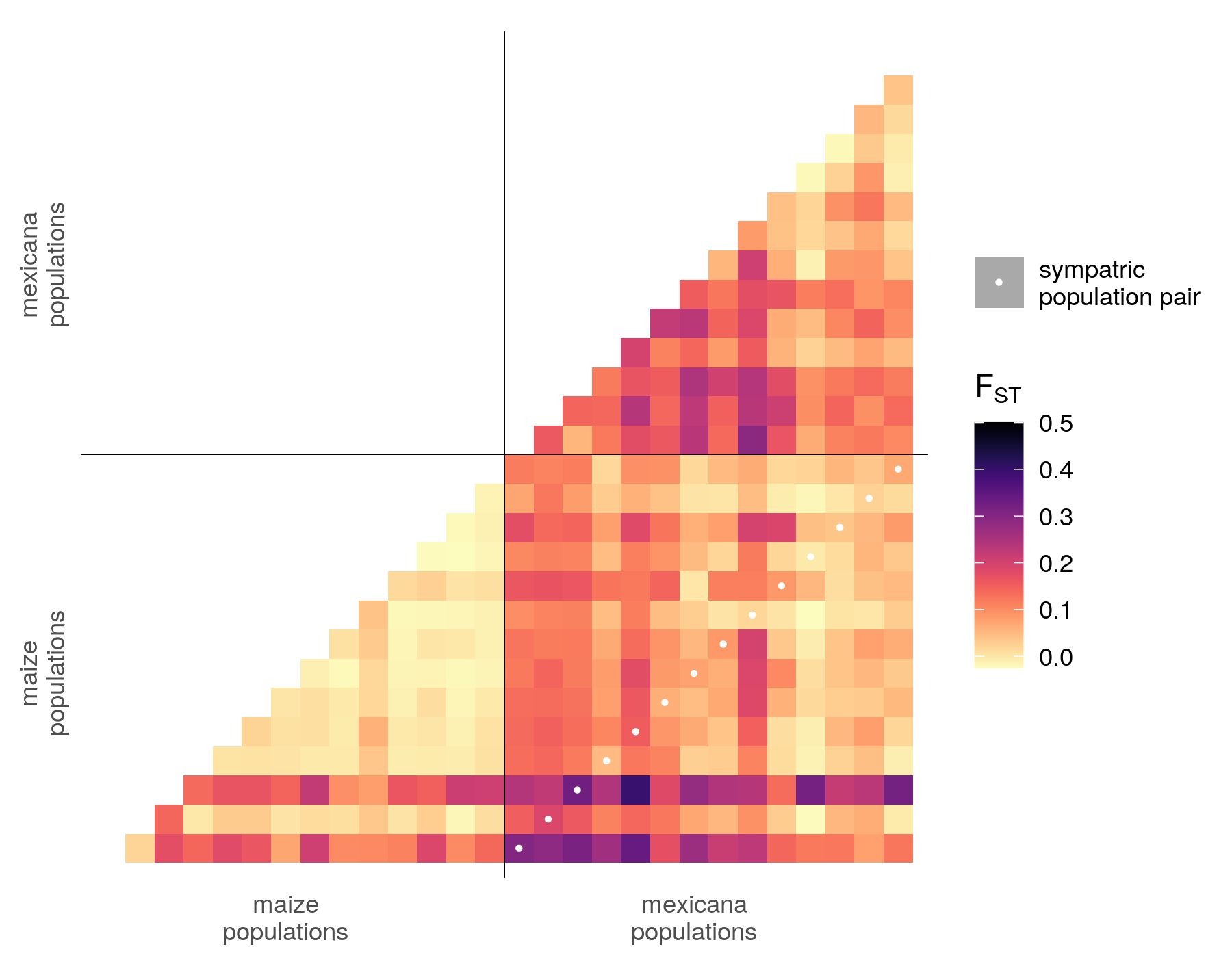

Supplement: S2 Fig — Pairwise FST between parviglumis ancestry tracts from population 1 (x-axis) and population 2 (y-axis). Populations are sorted by subspecies, then elevation. Local sympatric maize-mexicana population pairs are highlighted with a white dot and do not show reduced FST within parviglumis ancestry relative to other (non-local) maize-mexicana comparisons. (TIF) [file pgen.1009810.s009.tif]

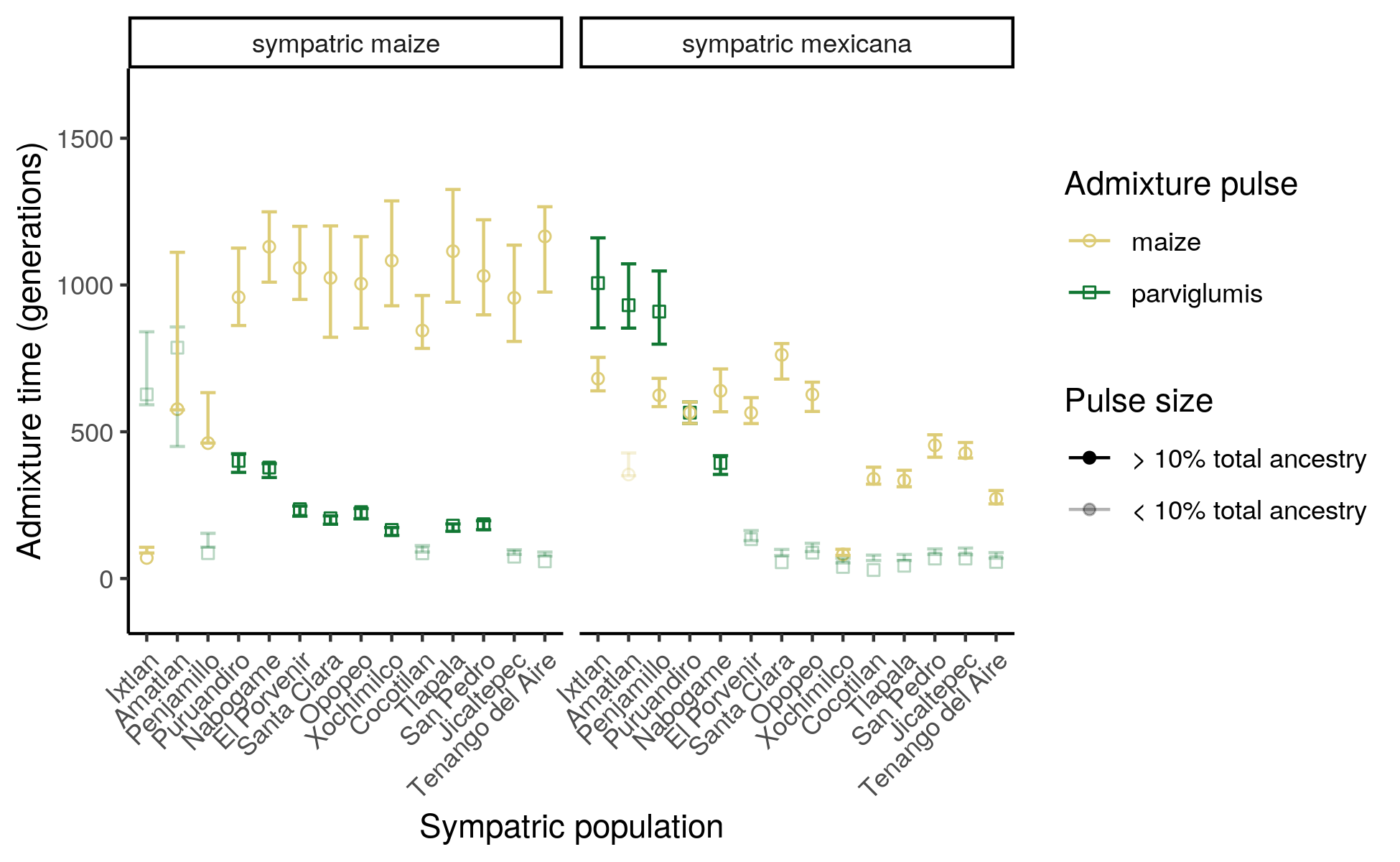

Supplement: S3 Fig — Estimated generations since admixture under a three-way admixture model: a founding mexicana population receives a pulse of parviglumis admixture and then a second pulse of maize admixture (possibly resulting in majority maize ancestry). Each sympatric maize and mexicana population was analyzed separately under this model, with 95% percentile confidence intervals for each admixture pulse based on 100 bootstrap samples of genomic blocks (1,000 SNPs per block). Estimates and bootstraps were produced during ancestry_hmm model fitting for local ancestry inference. Populations are ordered left to right by increasing elevation. Populations with very small genetic contributions from an ancestry pulse (< 10%) are faded because their timing estimates are less certain. (TIF) [file pgen.1009810.s010.tif]

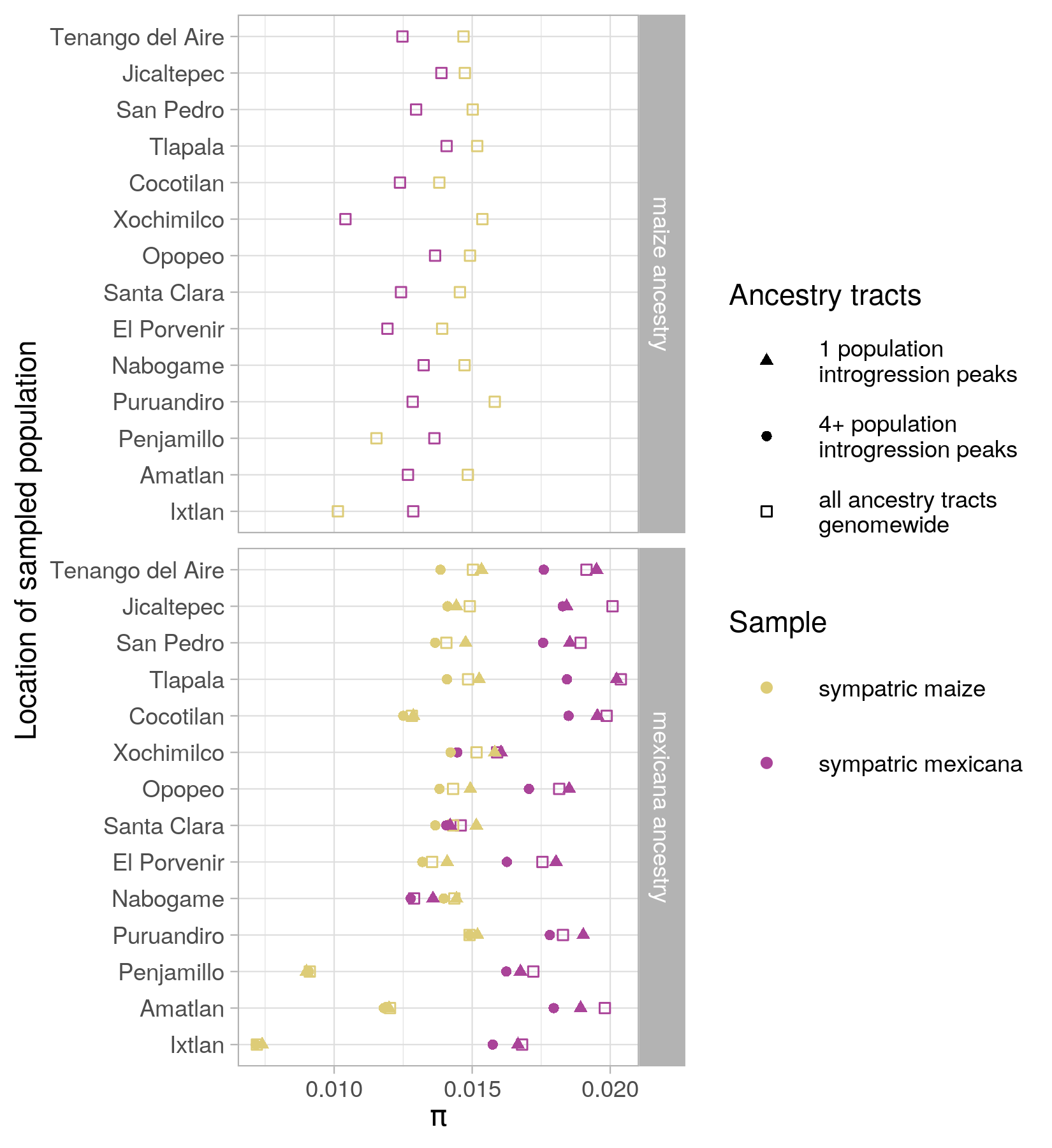

Supplement: S4 Fig — Each point summarises pairwise genetic diversity (π) for genomic regions with high-confidence homozygous maize or mexicana ancestry, calculated separately for the sympatric maize and mexicana populations at each sampled location. For maize ancestry (top), only a genomewide π is estimated, using all regions with high-confidence homozygous maize ancestry. For mexicana ancestry (bottom), π is calculated and plotted separately for three subsets of the genome: introgression peaks (> 2 s.d. above the mean) found in the focal maize population only, introgression peaks shared between the focal maize and at least 3 other maize populations, and a genomewide estimate. (TIF) [file pgen.1009810.s011.tif]

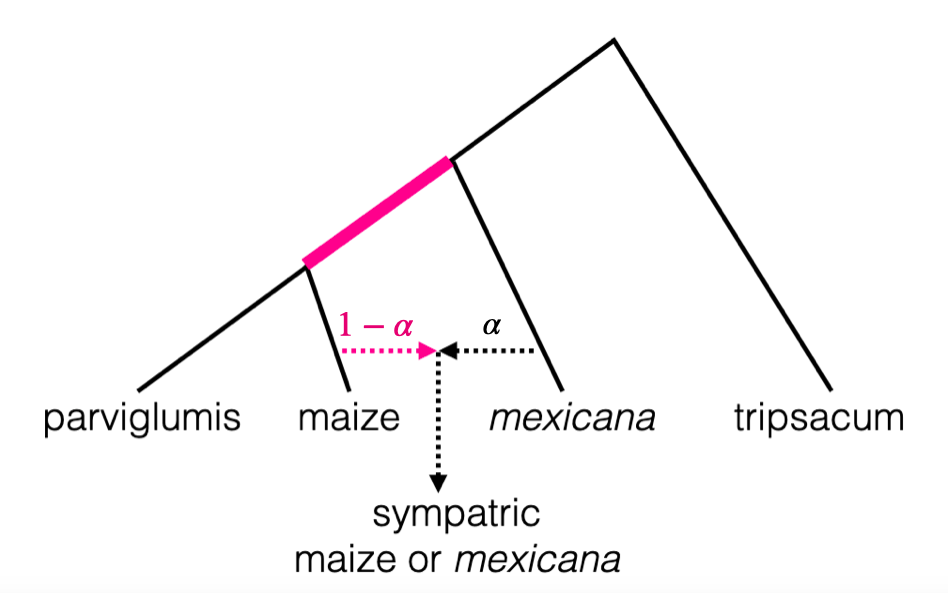

Supplement: S5 Fig — Phylogenetic tree assumed when estimating the ratio of f4 statistics. The pink branch represents the shared drift between maize and parviglumis that is introduced to the focal sympatric population via admixture of proportion 1 − α. We used only plants from the Amecameca site in our mexicana reference group for this analysis because that site showed no evidence of previous admixture. (TIF) [file pgen.1009810.s012.tif]

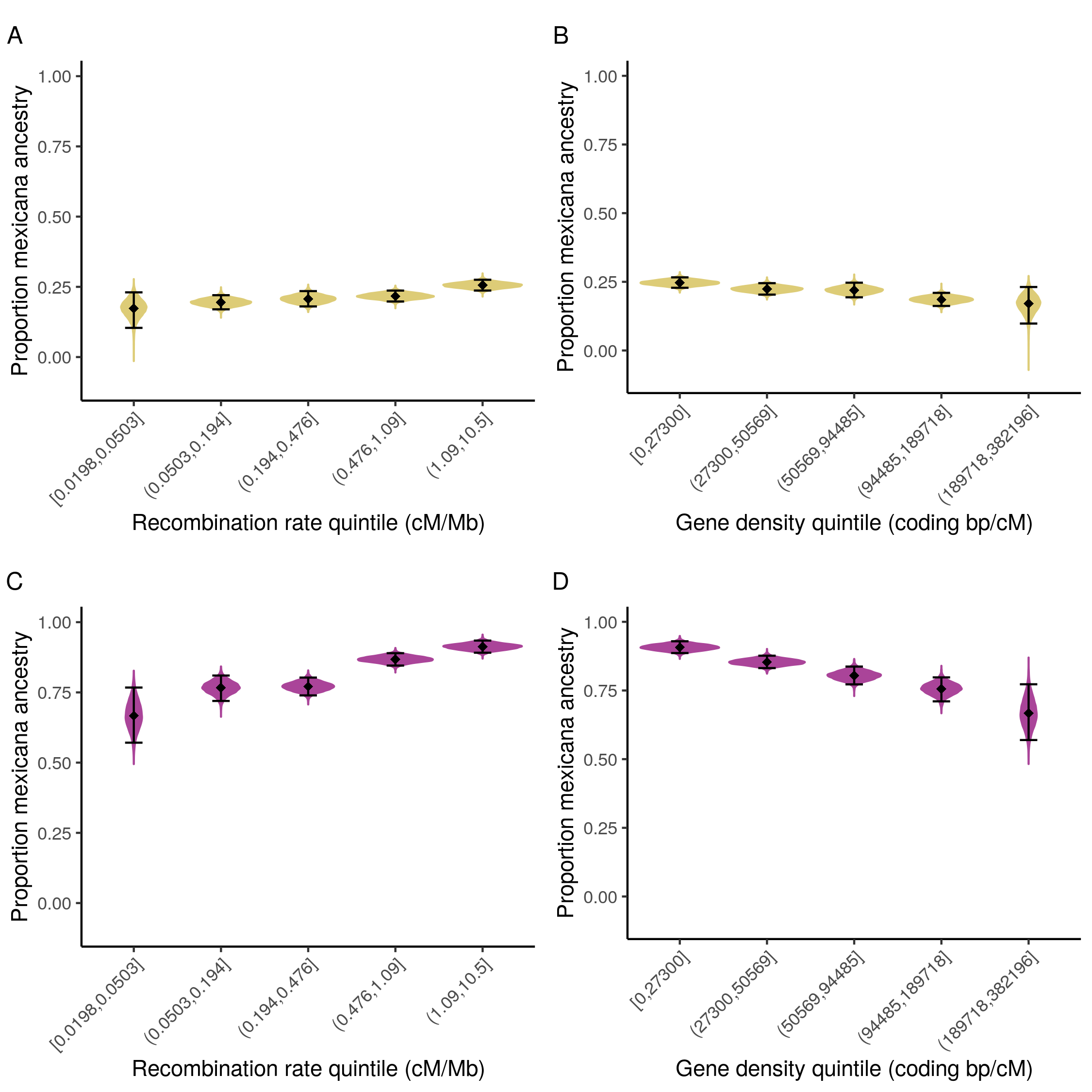

Supplement: S6 Fig — Mexicana ancestry proportions by genomic quintiles in sympatric maize (top) and mexicana (bottom), estimated using f4 ratios. Spearman’s rank correlations for each plot: (A) Mexicana ancestry by recombination rate quintile in sympatric maize (ρ = 1.00, CI95[0.30, 1.00]), (B) mexicana ancestry by gene density (coding bp/cM) quintile in symaptric maize (ρ = −1.00, CI95[−1.00, −0.40]), (C) Mexicana ancestry by recombination rate quintile in sympatric mexicana (ρ = 1.00, CI95[0.70, 1.00]), and (D) mexicana ancestry by gene density (coding bp/cM) quintile in symaptric mexicana (ρ = −1.00, CI95[−1.00, −0.90]). Mean ancestry per quintile and 95% percentile bootstrap confidence interval (n = 10,000) are depicted in black. Violin plots show the density of ancestry estimates for individual bootstraps re-sampled within quintiles. Note: Ancestry estimates from f4’s are less reliable for sympatric mexicana than sympatric maize because of significant parviglumis ancestry in some sympatric mexicana populations. The f4 ratio estimation method assumes no additional unmodeled admixture on the population tree (see S5 Fig). (TIF) [file pgen.1009810.s013.tif]

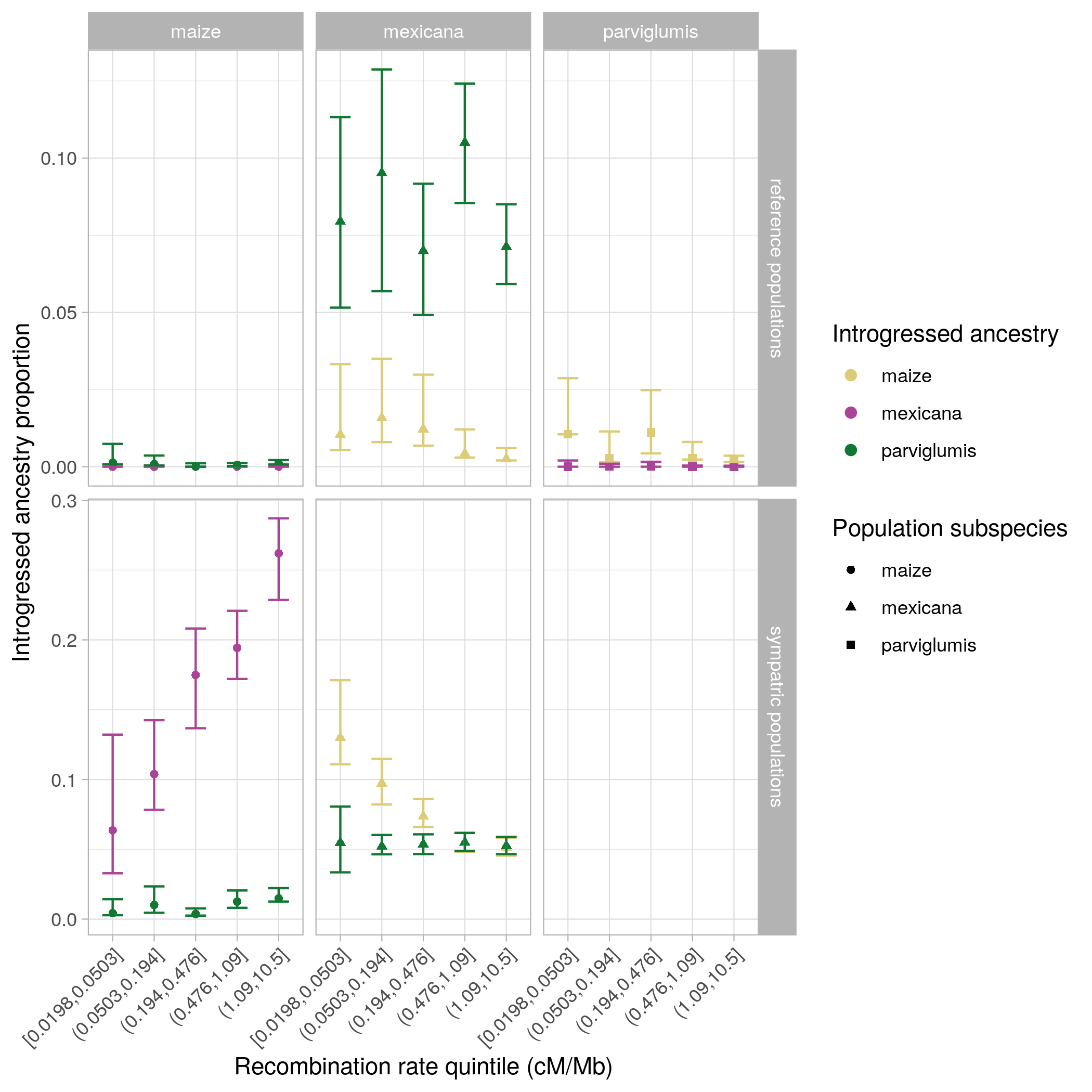

Supplement: S7 Fig — Inferred introgressed ancestry in reference populations (top) and sympatric maize and mexicana populations (bottom) using NGSAdmix (K = 3) by recombination rate quintiles. Group mean and 95% percentile bootstrap confidence interval (n = 100) are shown. Different colors distinguish the different introgressing ancestries, and different shapes distinguish the Zea subspecies for the sampled populations. (TIF) [file pgen.1009810.s014.tif]

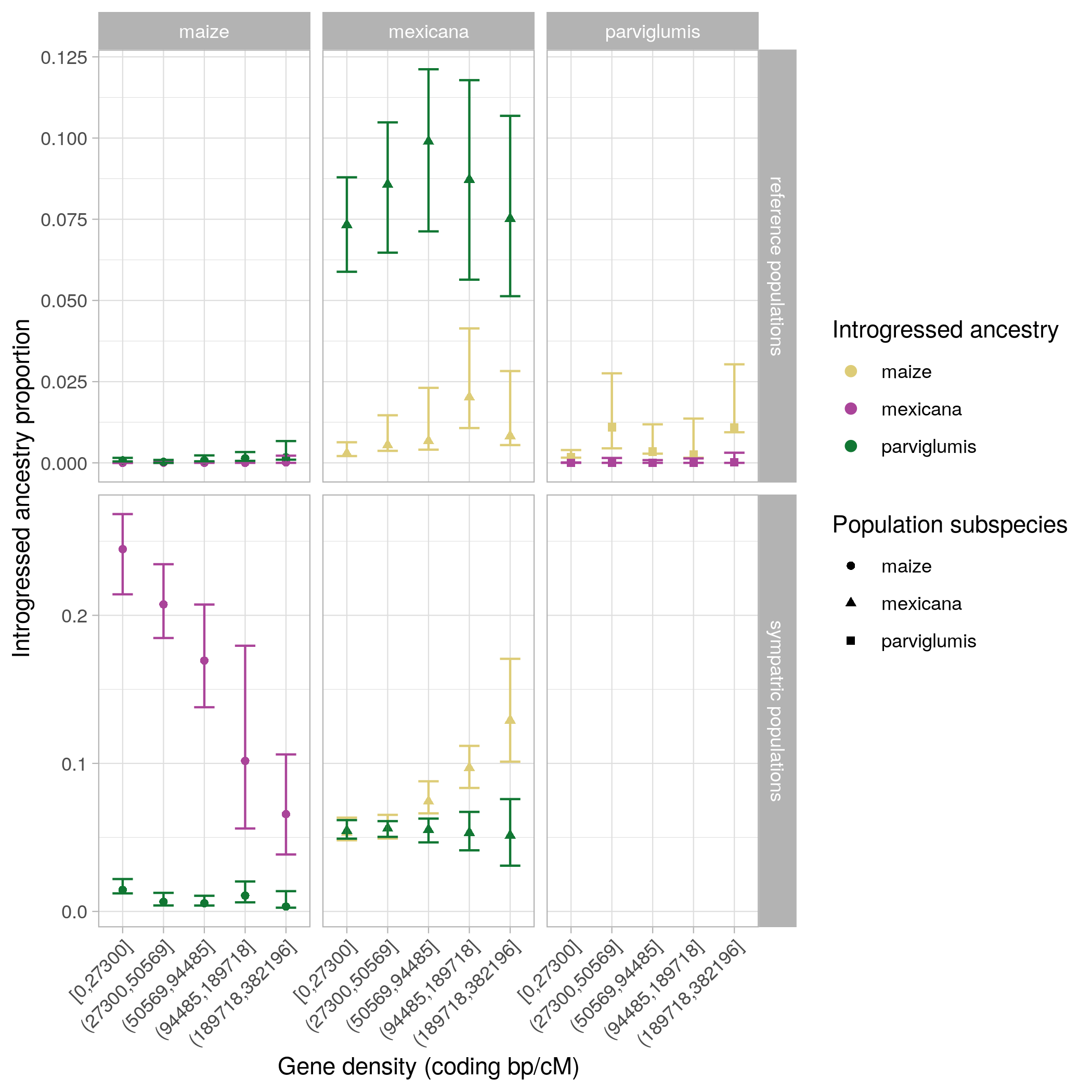

Supplement: S8 Fig — Inferred introgressed ancestry in reference populations (top) and sympatric maize and mexicana populations (bottom) using NGSAdmix (K = 3) by coding density quintiles. Group mean and 95% percentile bootstrap confidence interval (n = 100) are shown. Different colors distinguish the different introgressing ancestries, and different shapes distinguish the Zea subspecies for the sampled populations. (TIF) [file pgen.1009810.s015.tif]

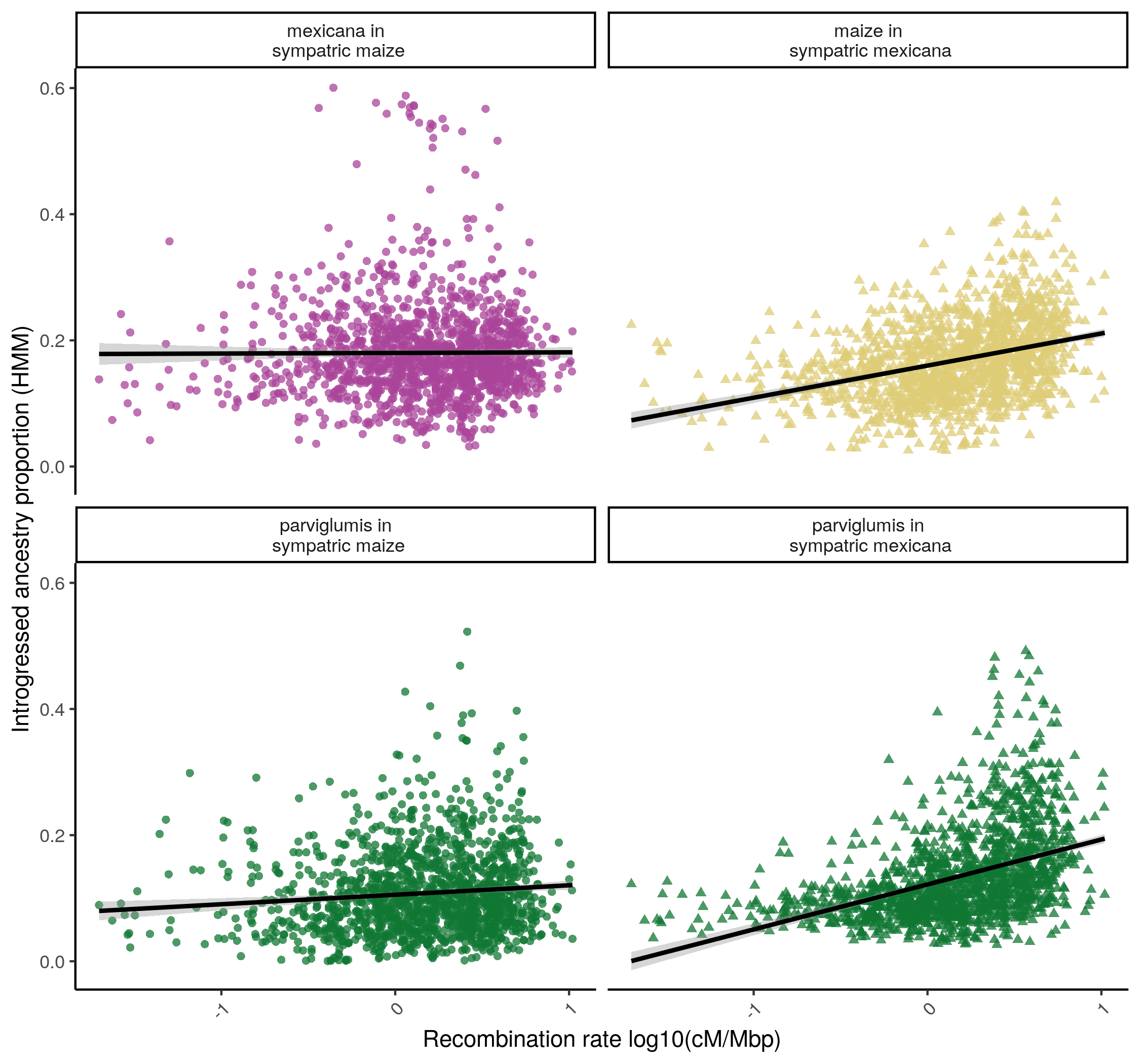

Supplement: S9 Fig — Estimated local ancestry in sympatric maize and mexicana samples using ancestry_hmm. Each point is a 1 cM genomic window and the line shows the best linear model fit for mean introgressed ancestry by recombination rate on a log scale. Different colors distinguish the different introgressing ancestries, and different shapes distinguish the Zea subspecies for the sampled sympatric populations. (TIF) [file pgen.1009810.s016.tif]

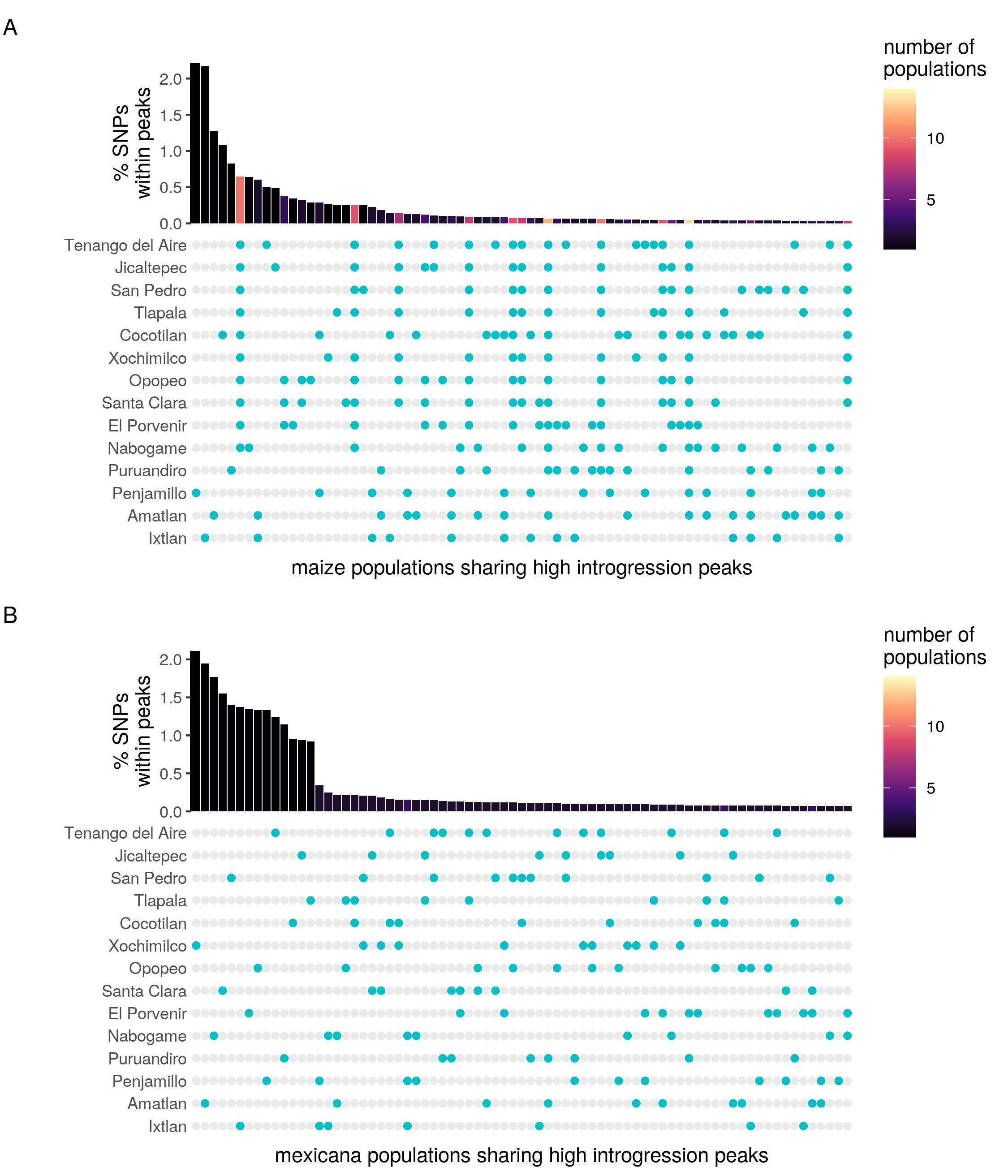

Supplement: S10 Fig — Here we show the 75 most common combinations of populations that share ancestry peaks (introgressed ancestry > 2 s.d. above each population’s mean ancestry) for (A) sympatric maize and (B) sympatric mexicana. Bar height represents the percent of SNPs genomewide within peaks shared by the populations highlighted in blue below. Only for sympatric maize do we find that larger sets of populations (brighter colored bars) commonly share peaks across the genome. Populations are ordered from high to low elevation (top to bottom), showing that introgression peak sharing is more common among combinations of the highest elevation maize populations. (TIF) [file pgen.1009810.s017.tif]

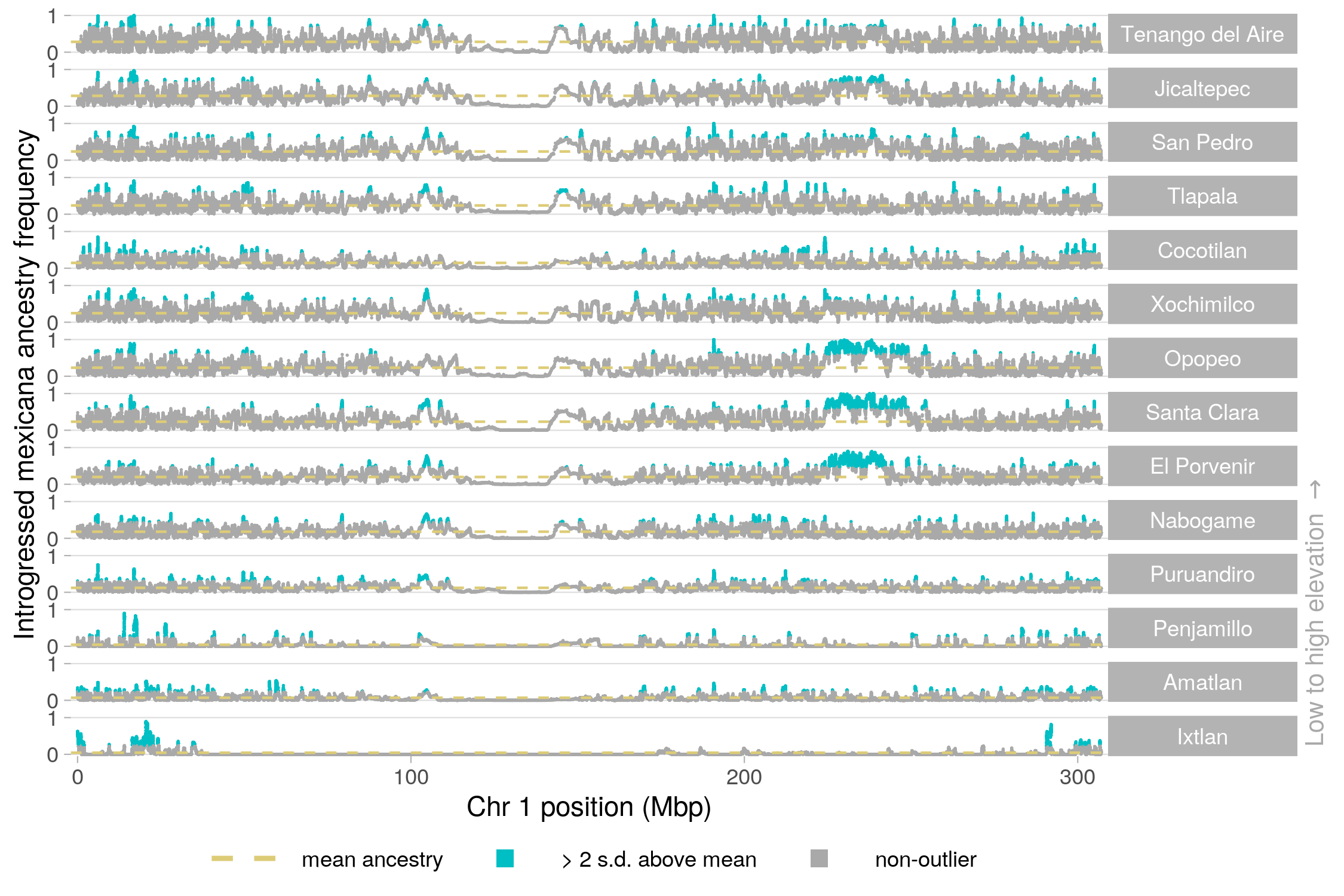

Supplement: S11 Fig — (TIF) [file pgen.1009810.s018.tif]

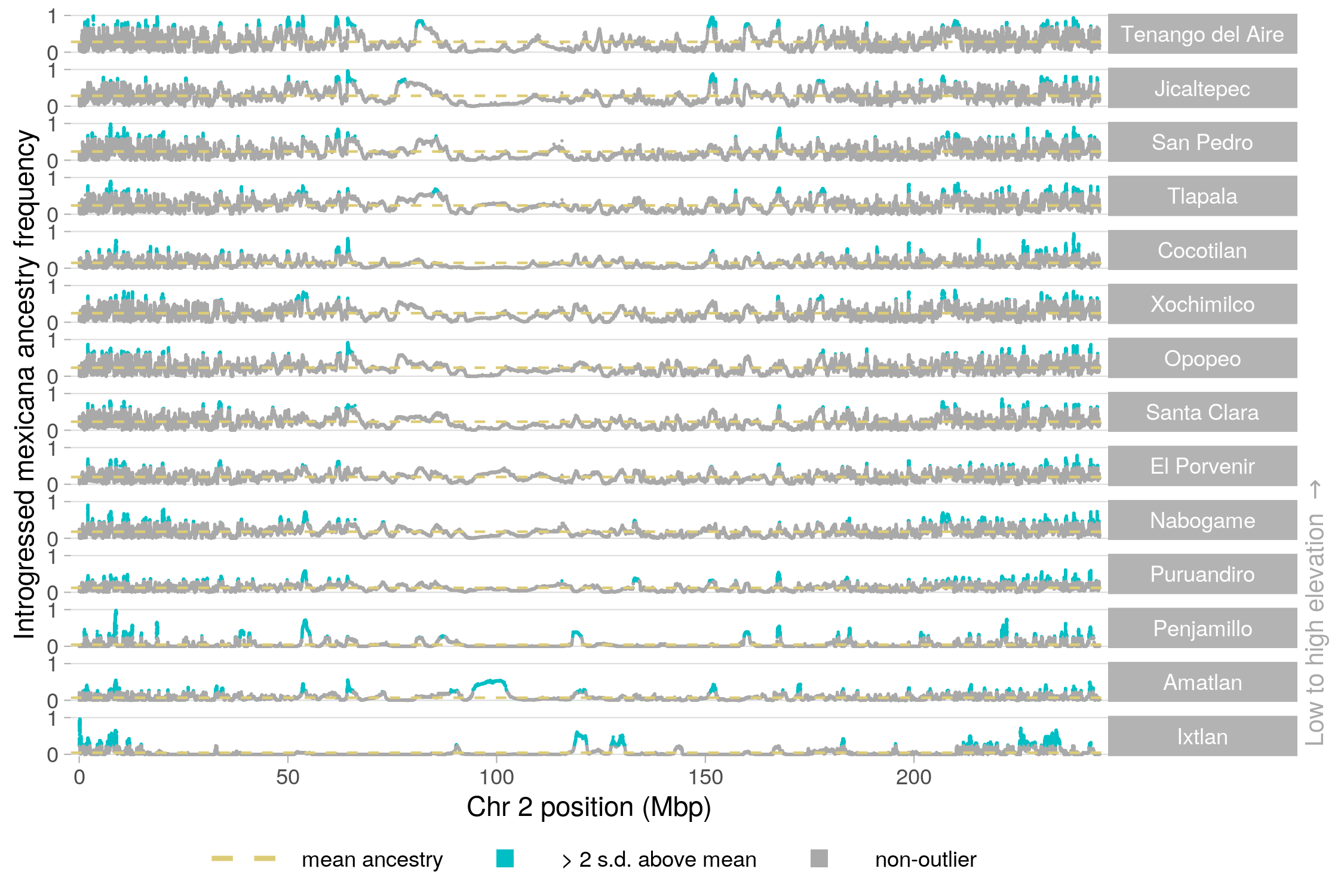

Supplement: S12 Fig — (TIF) [file pgen.1009810.s019.tif]

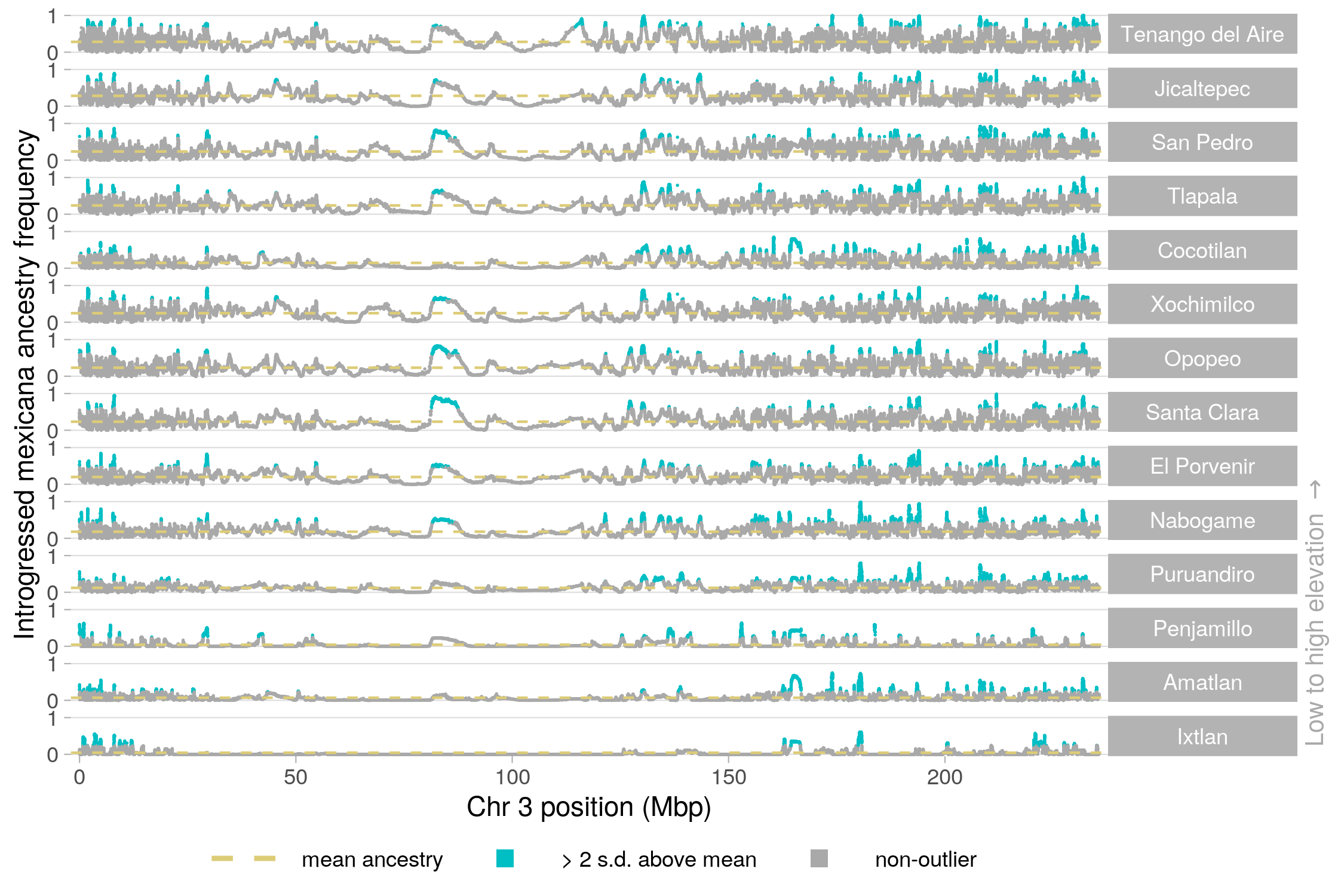

Supplement: S13 Fig — (TIF) [file pgen.1009810.s020.tif]

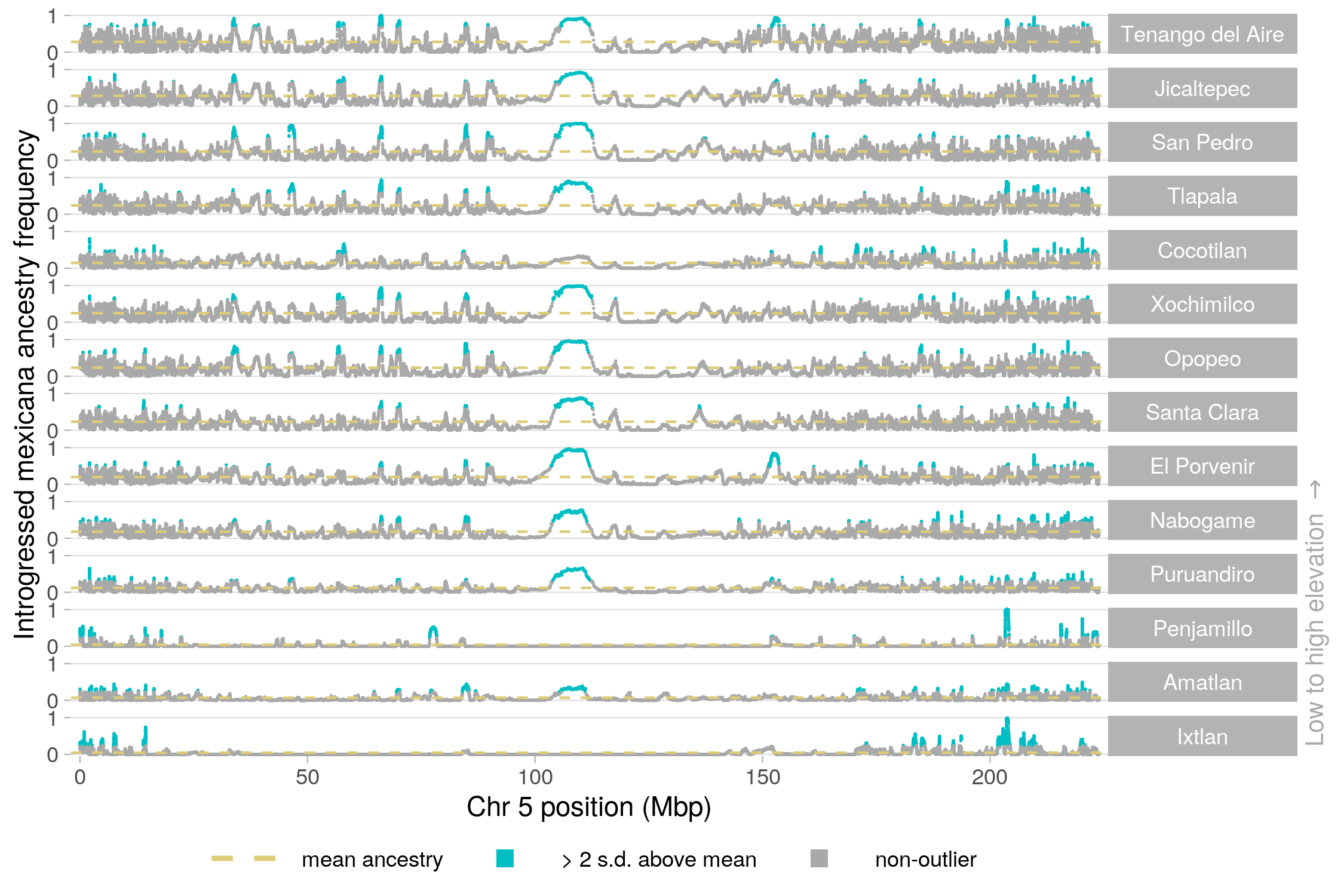

Supplement: S14 Fig — (TIF) [file pgen.1009810.s021.tif]

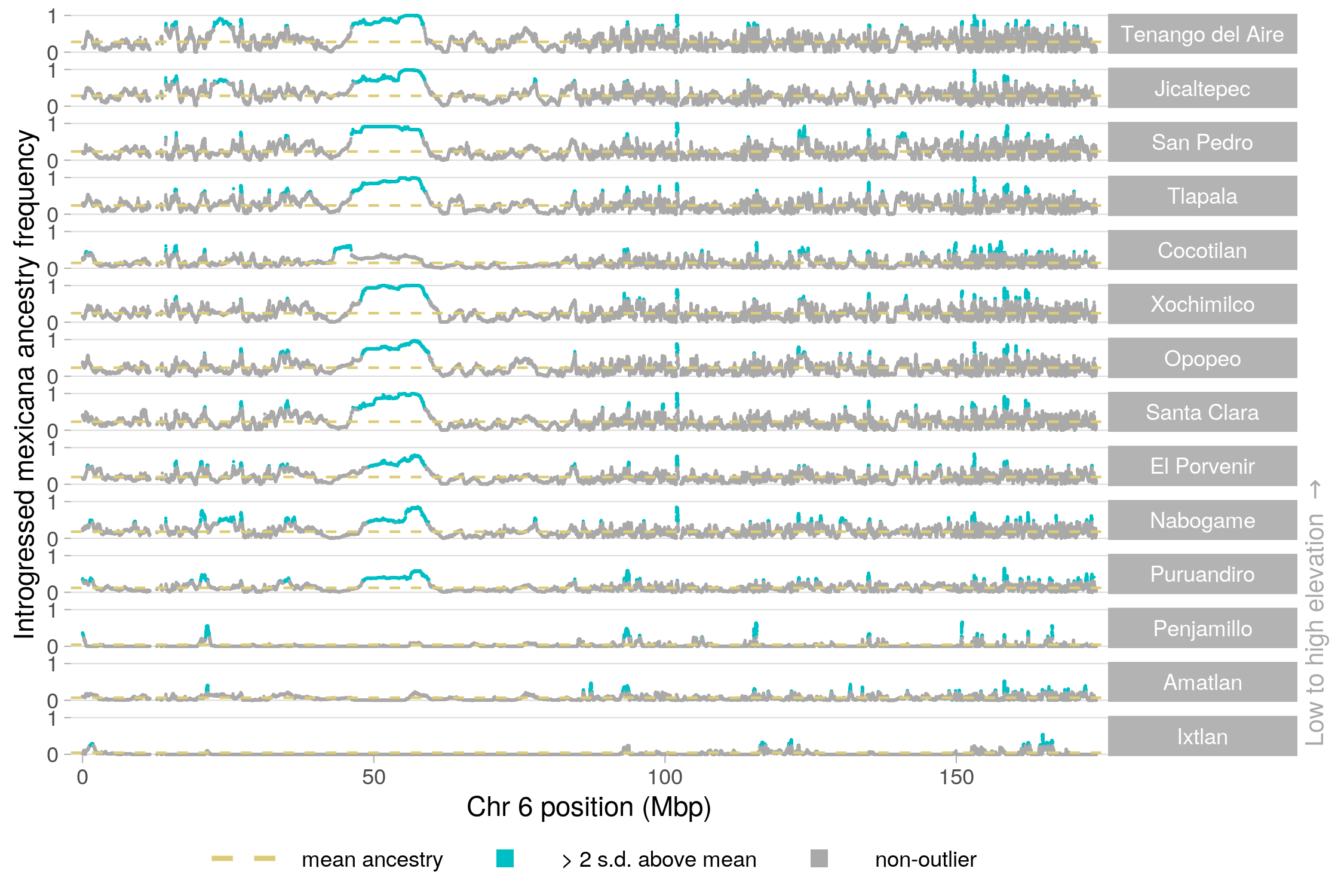

Supplement: S15 Fig — (TIF) [file pgen.1009810.s022.tif]

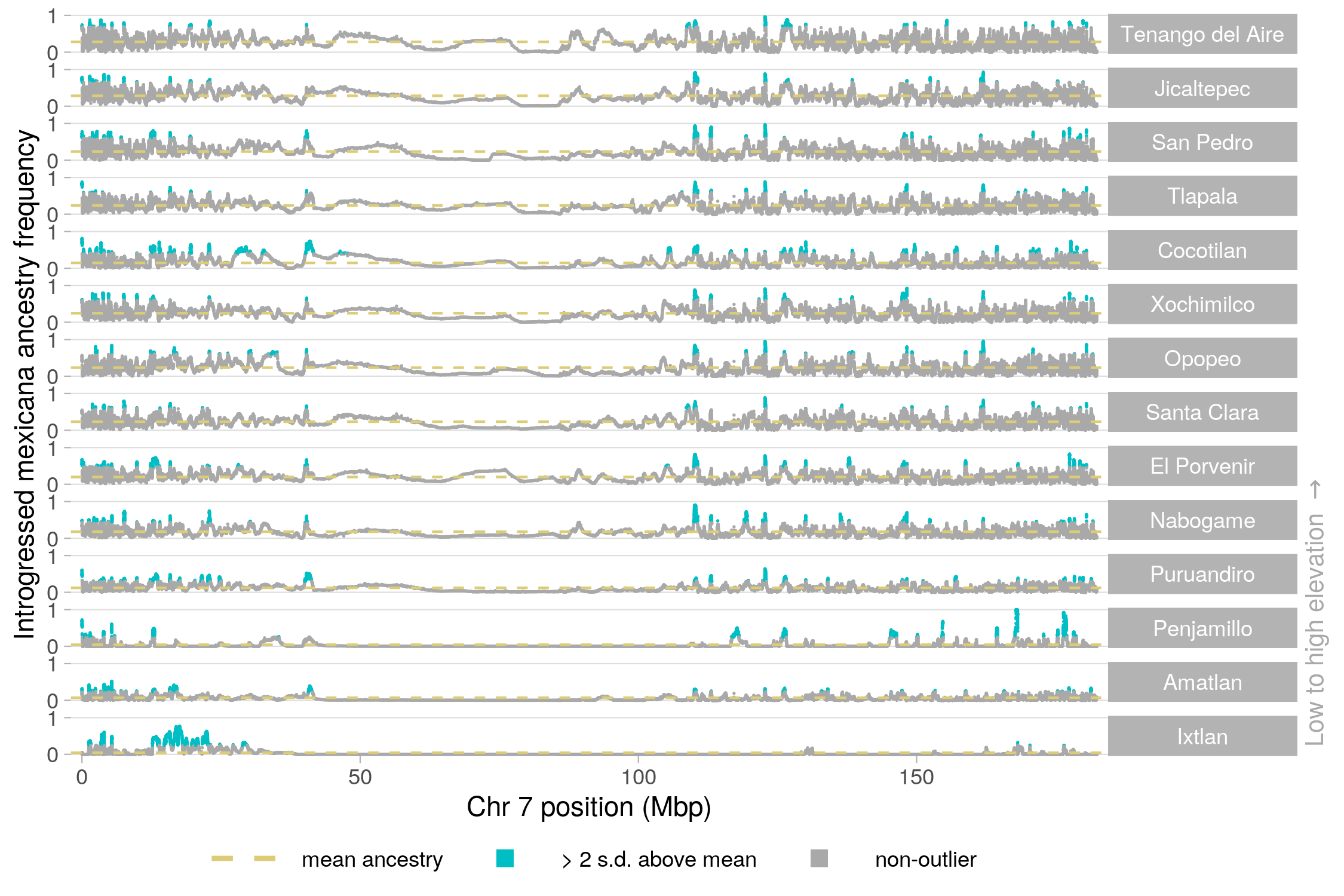

Supplement: S16 Fig — (TIF) [file pgen.1009810.s023.tif]

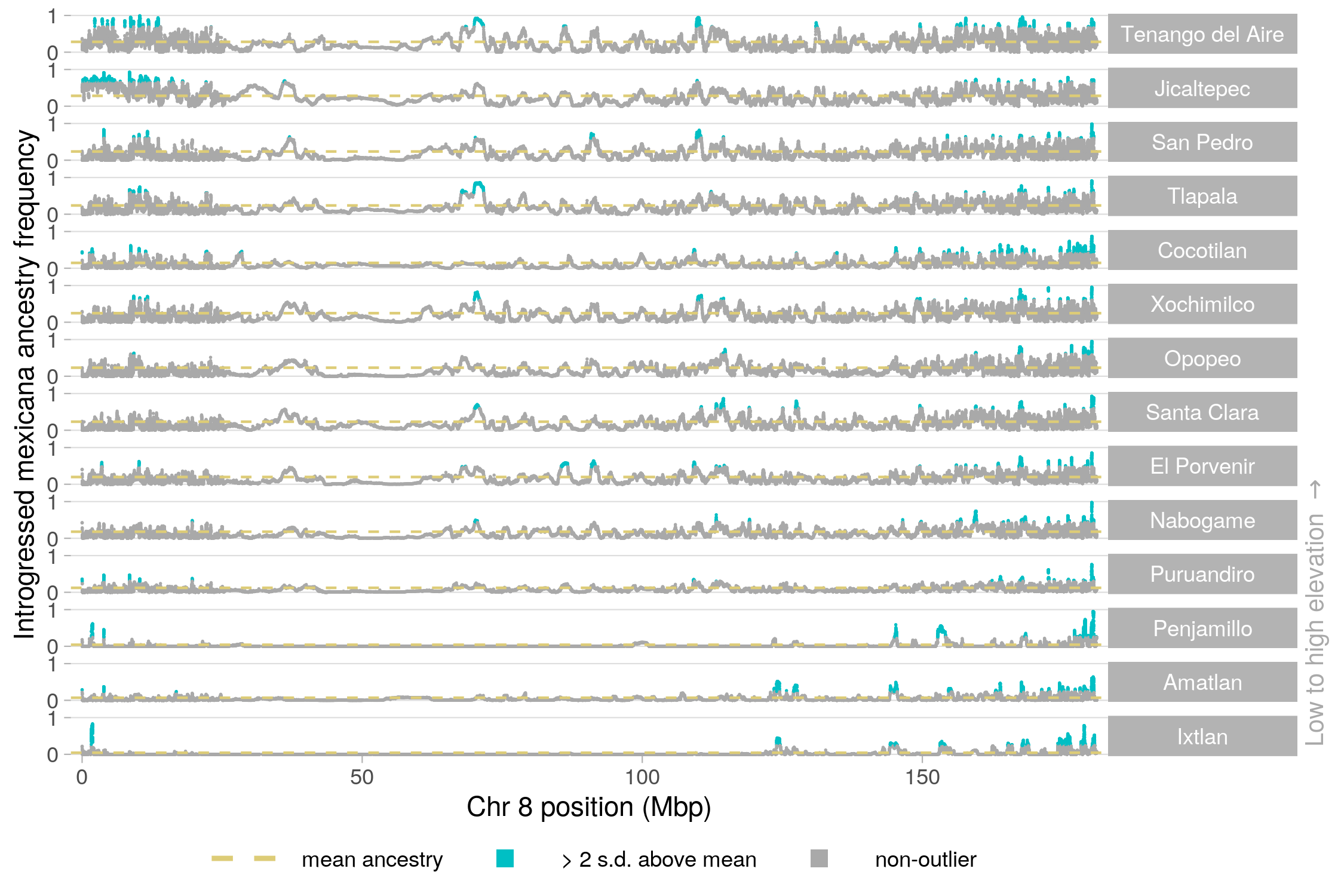

Supplement: S17 Fig — (TIF) [file pgen.1009810.s024.tif]

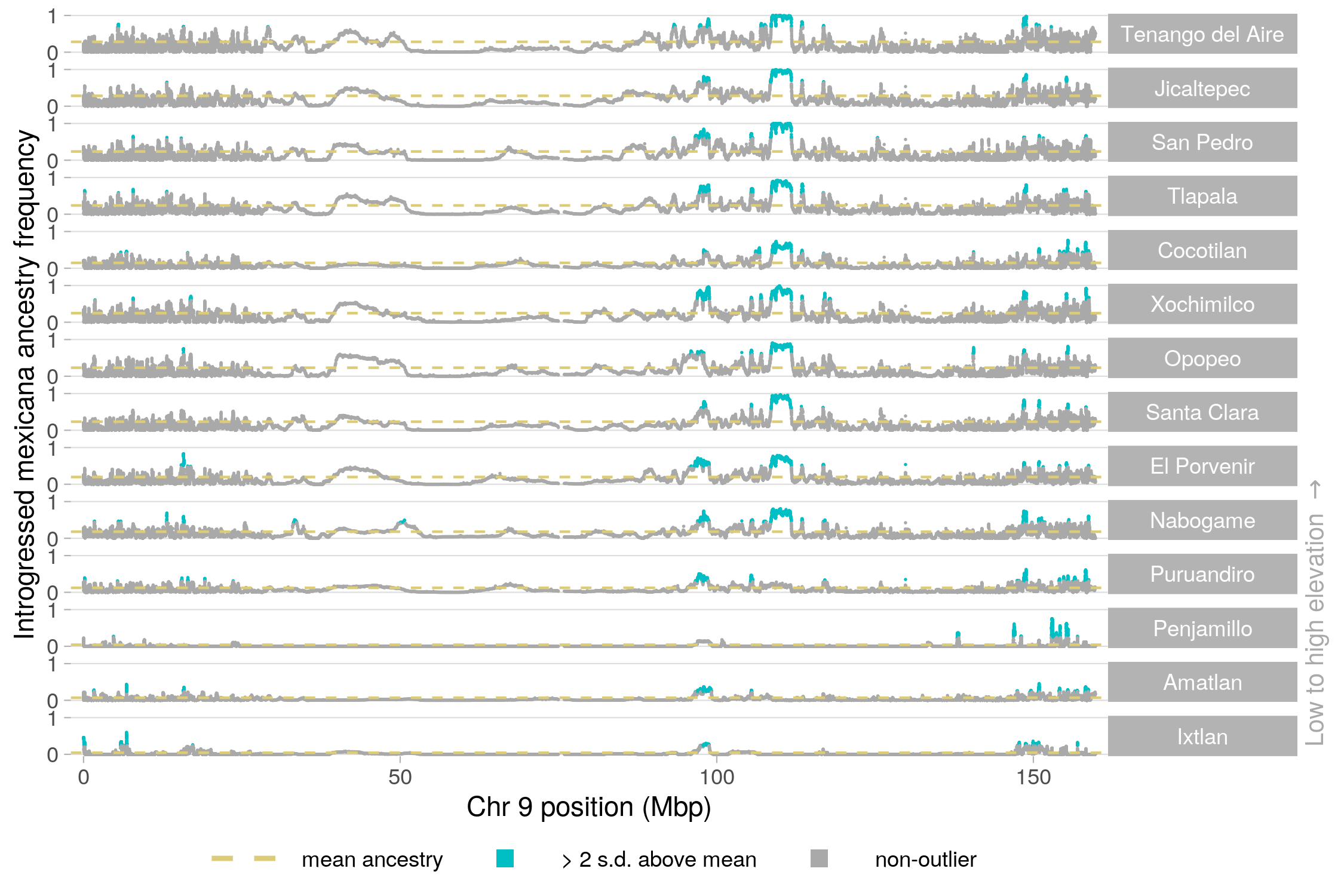

Supplement: S18 Fig — (TIF) [file pgen.1009810.s025.tif]

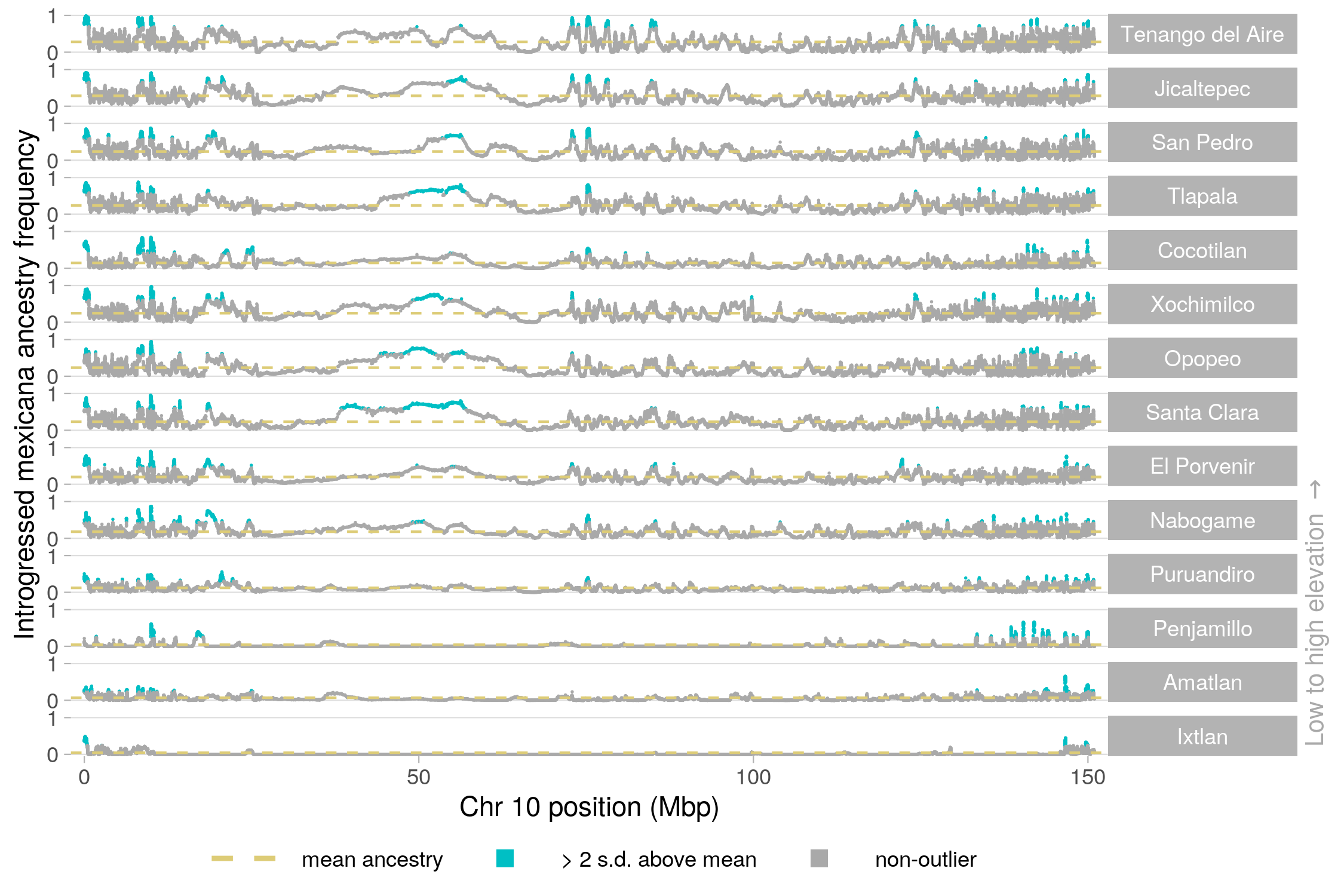

Supplement: S19 Fig — (TIF) [file pgen.1009810.s026.tif]

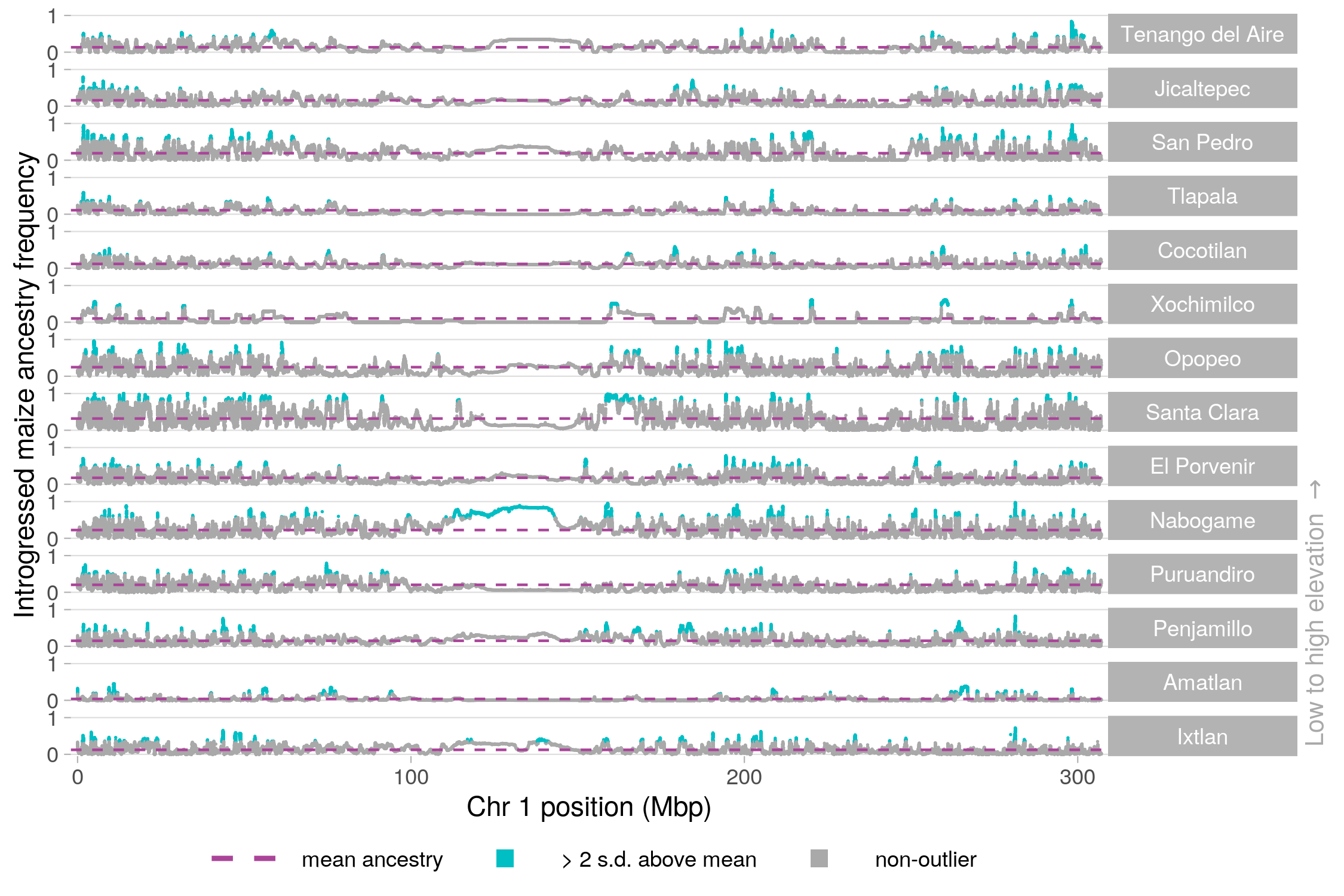

Supplement: S20 Fig — (TIF) [file pgen.1009810.s027.tif]

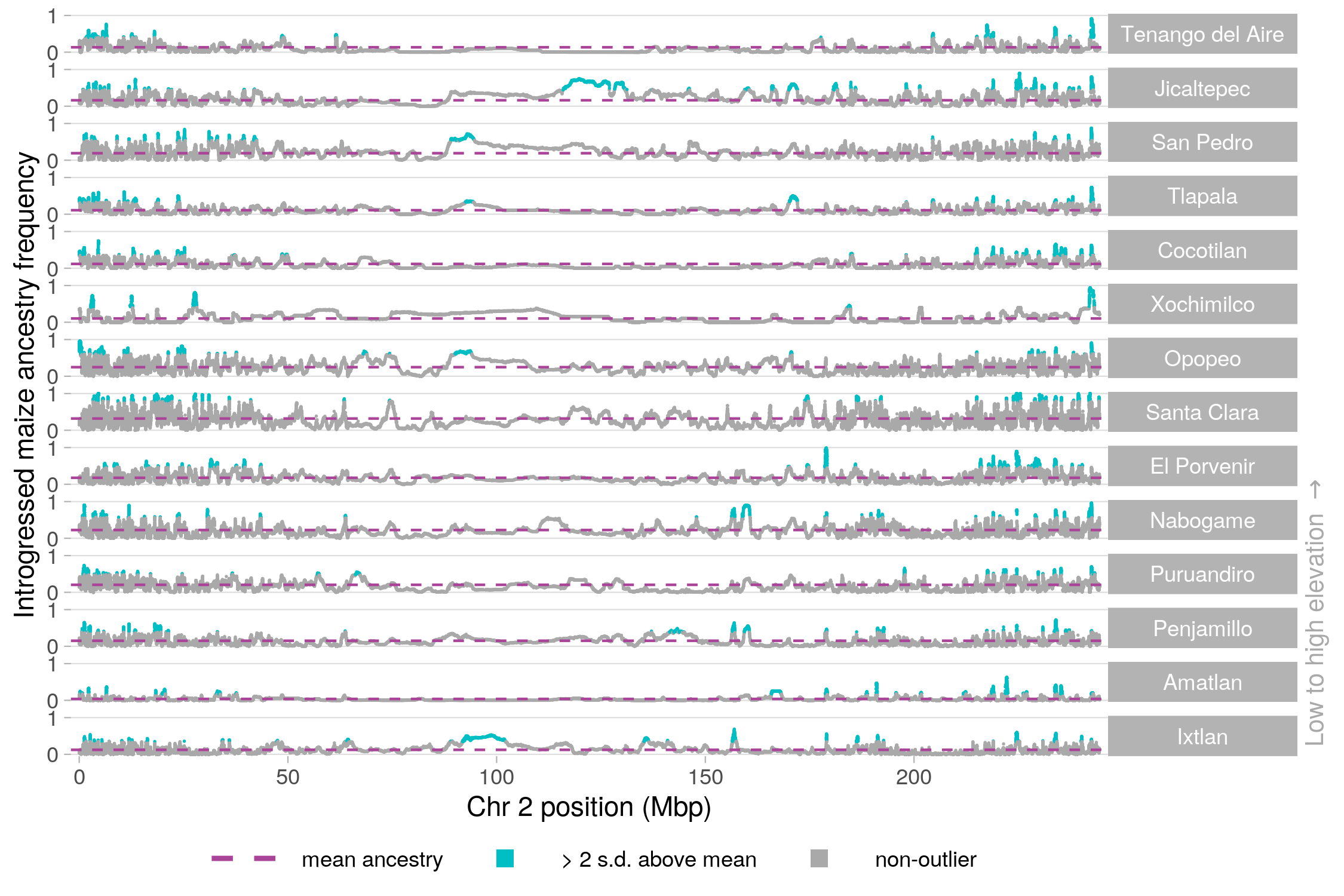

Supplement: S21 Fig — (TIF) [file pgen.1009810.s028.tif]

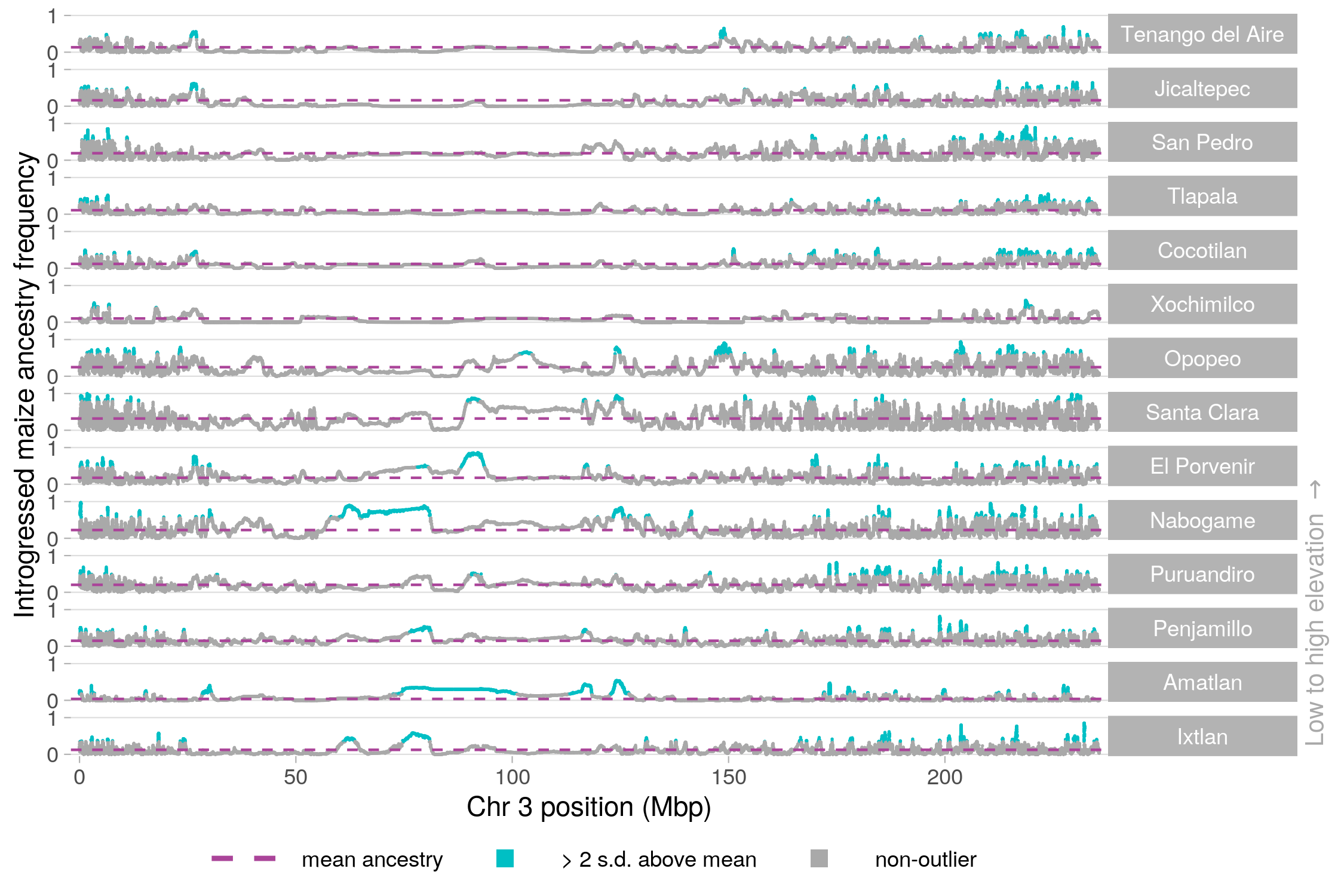

Supplement: S22 Fig — (TIF) [file pgen.1009810.s029.tif]

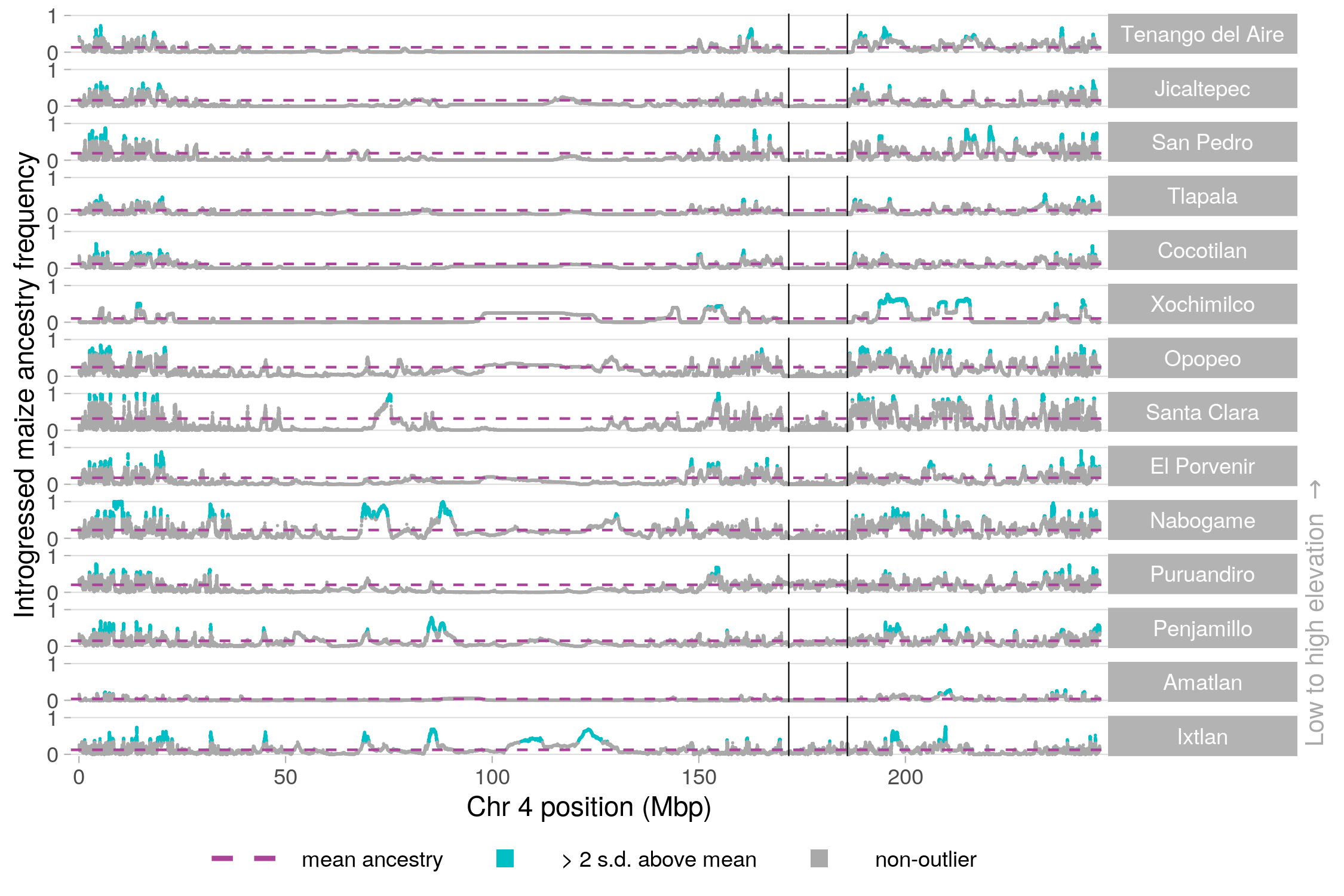

Supplement: S23 Fig — Vertical lines indicate the coordinates for Inv4m. (TIF) [file pgen.1009810.s030.tif]

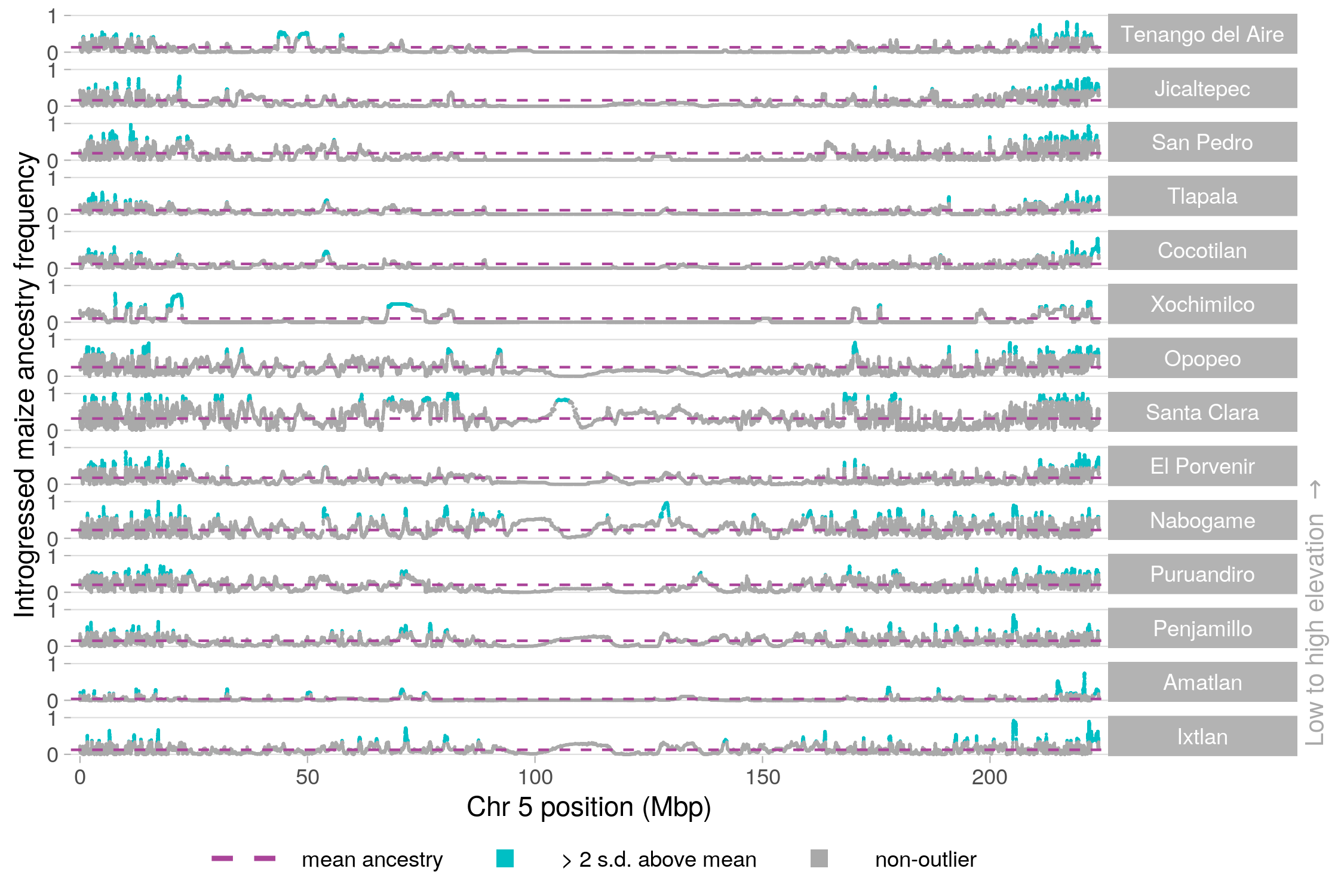

Supplement: S24 Fig — (TIF) [file pgen.1009810.s031.tif]

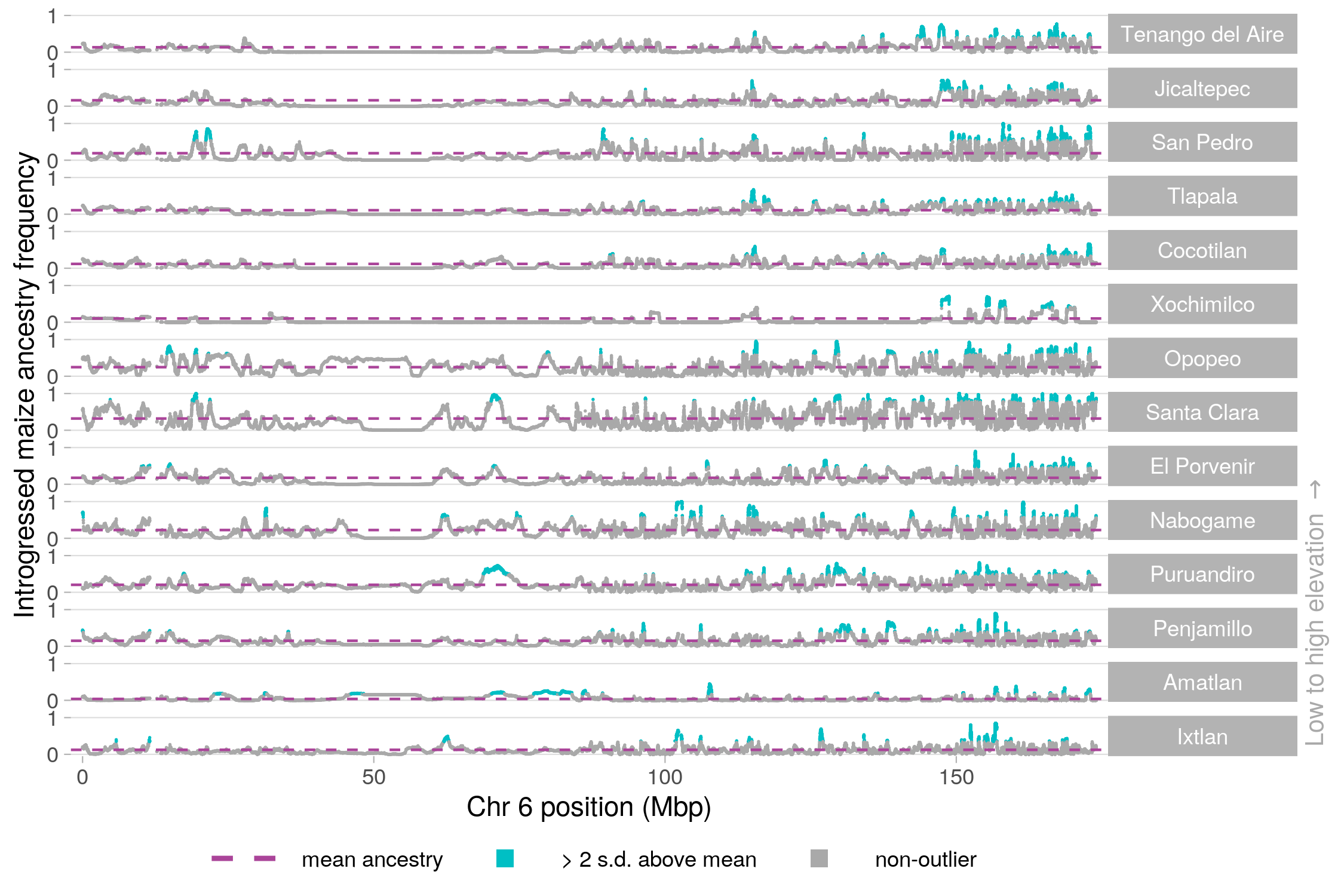

Supplement: S25 Fig — (TIF) [file pgen.1009810.s032.tif]

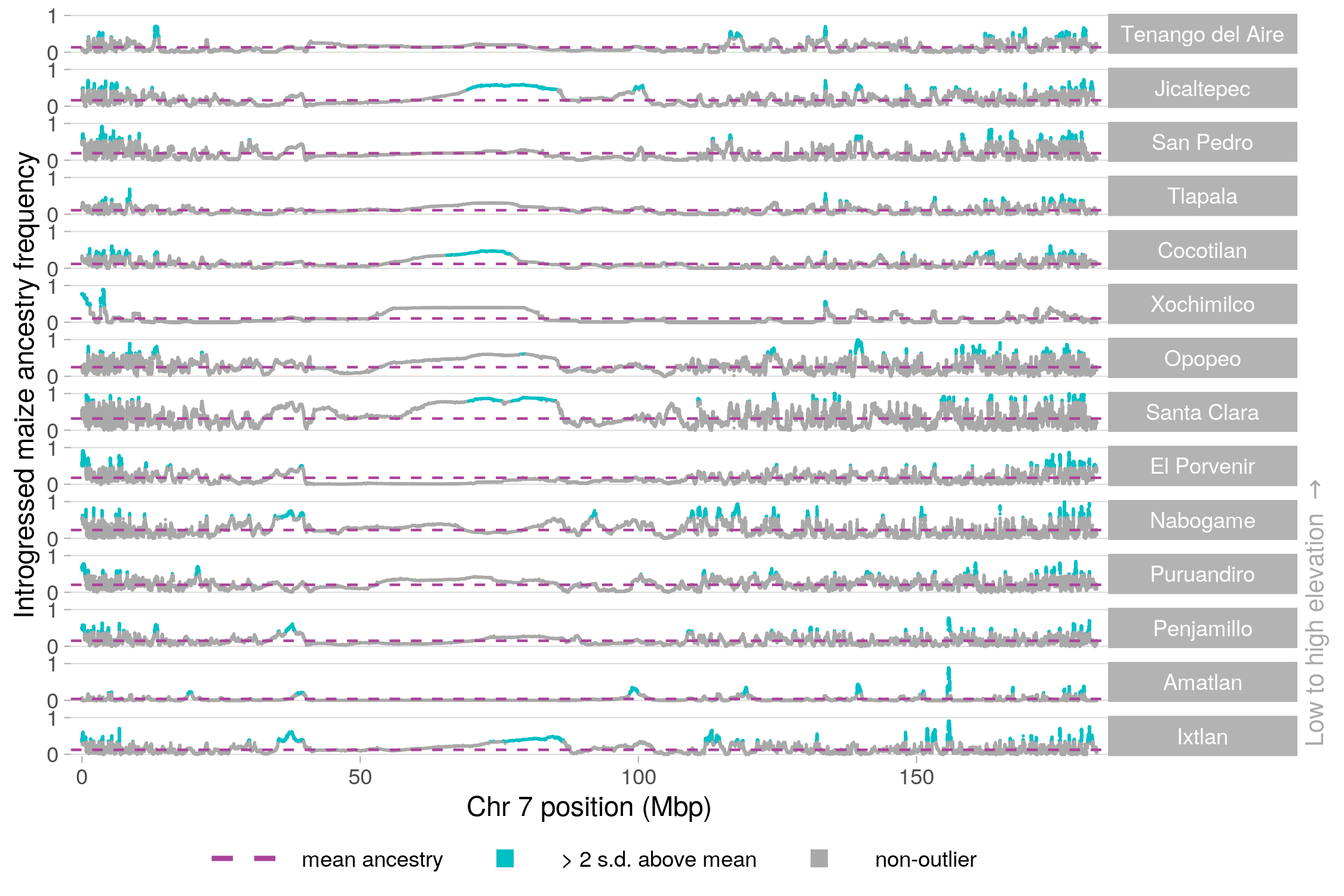

Supplement: S26 Fig — (TIF) [file pgen.1009810.s033.tif]

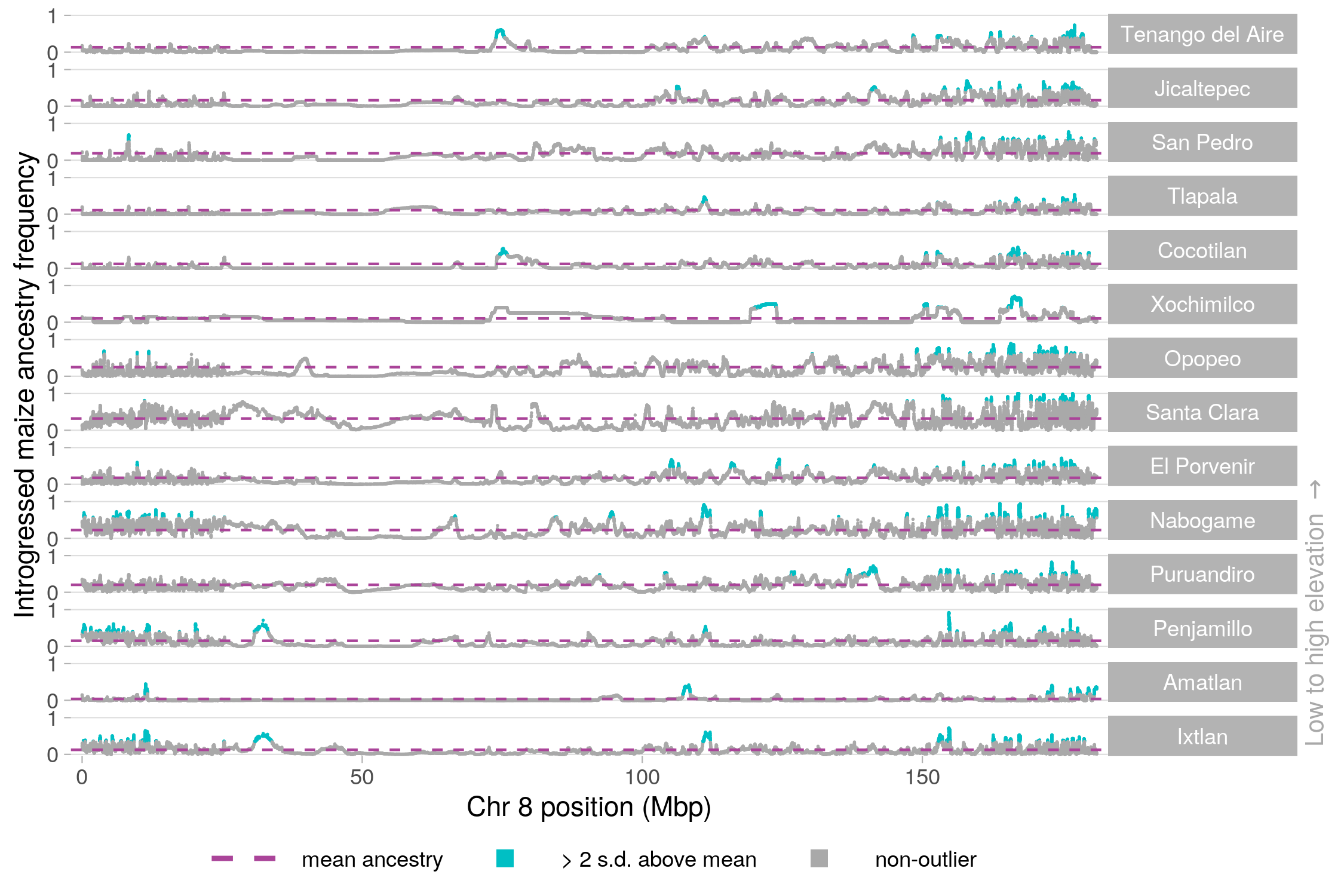

Supplement: S27 Fig — (TIF) [file pgen.1009810.s034.tif]

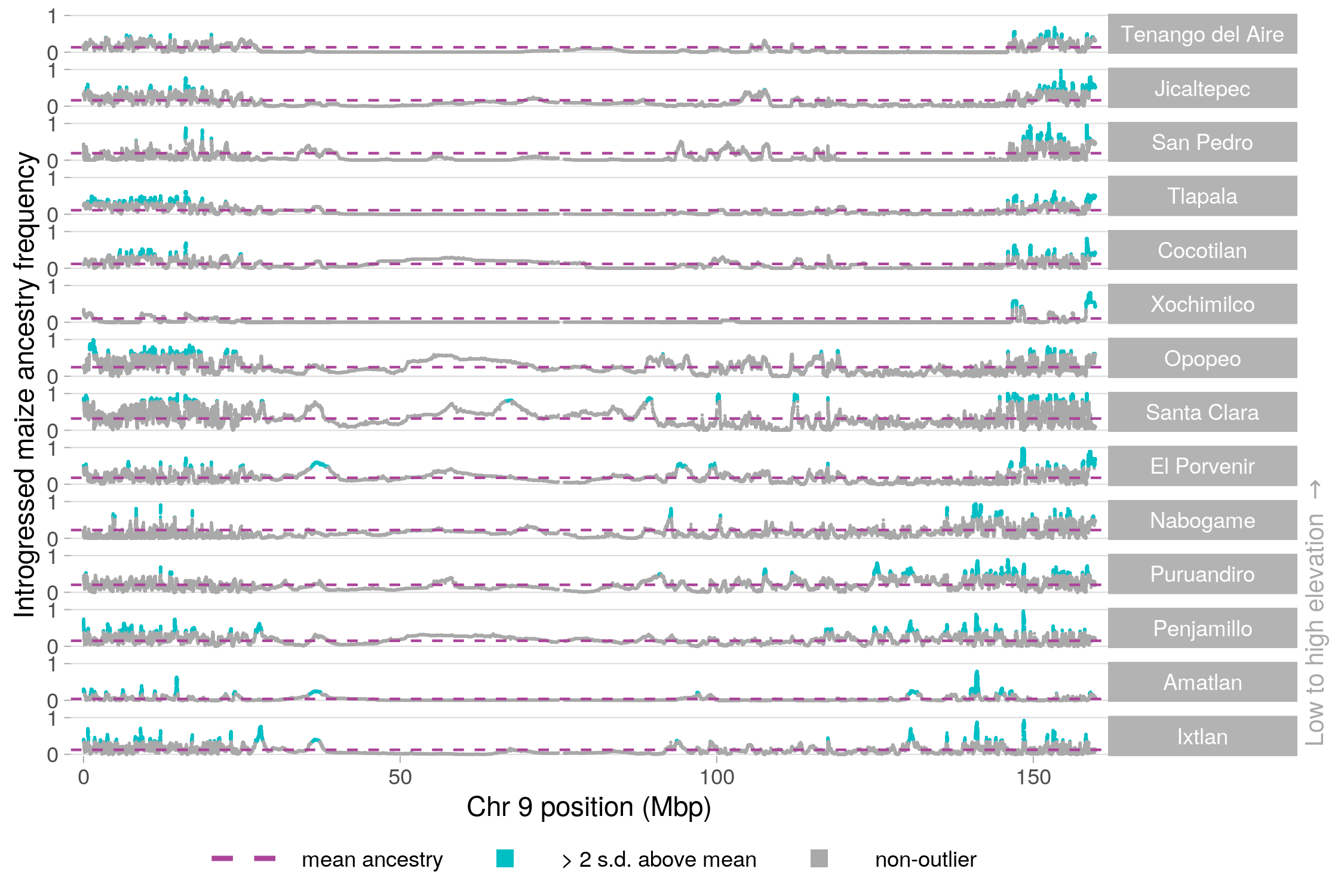

Supplement: S28 Fig — (TIF) [file pgen.1009810.s035.tif]

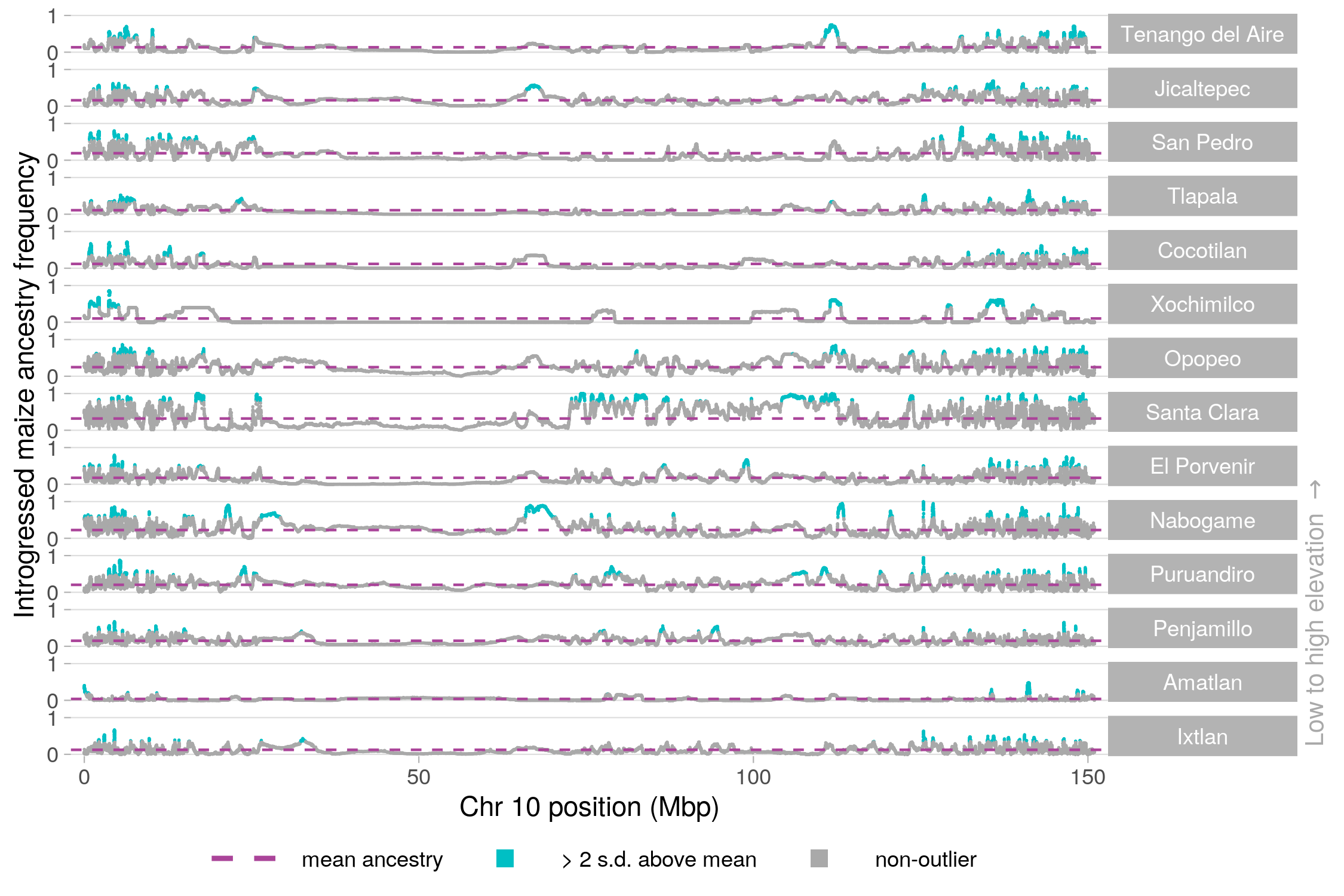

Supplement: S29 Fig — (TIF) [file pgen.1009810.s036.tif]

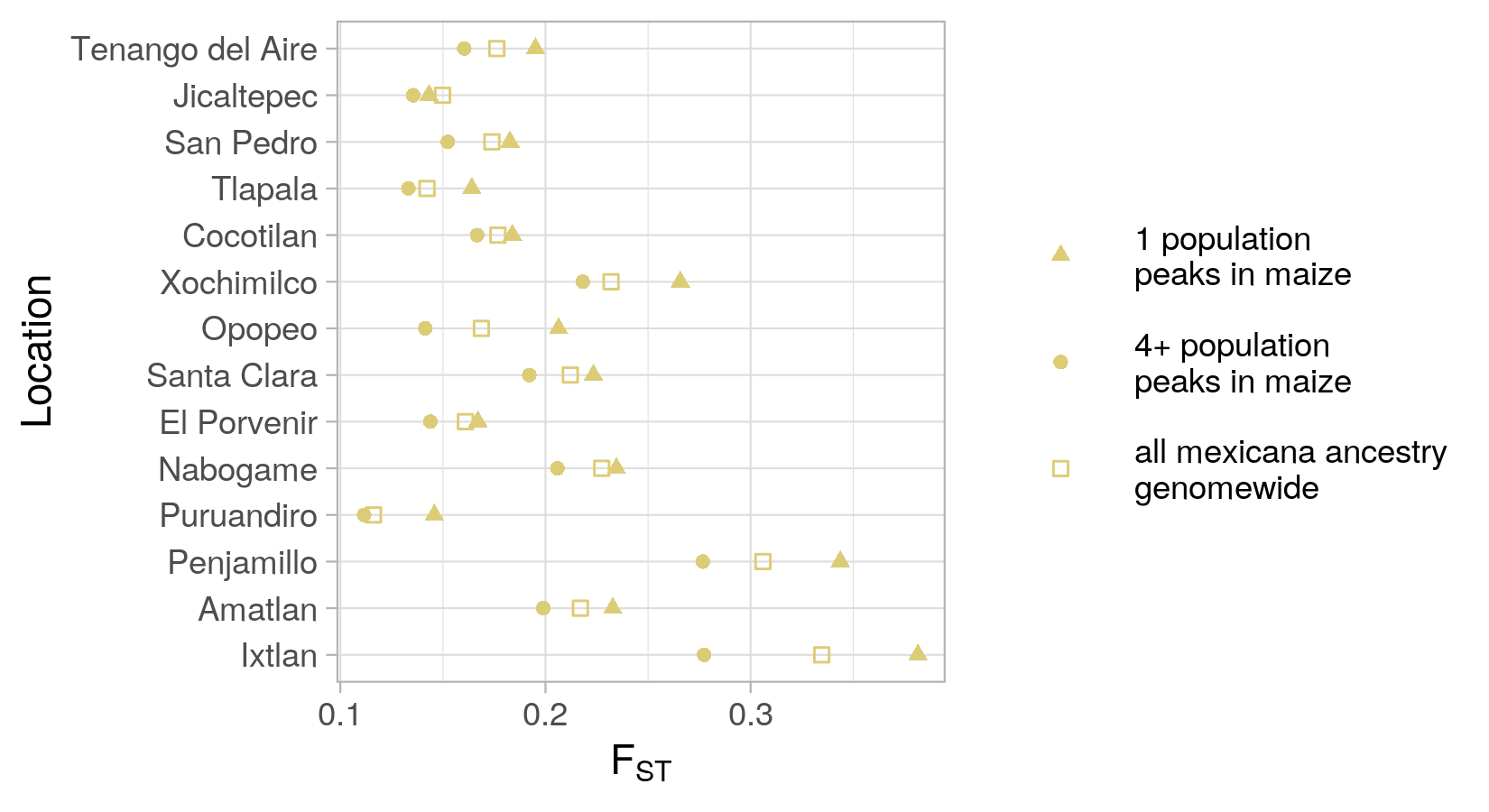

Supplement: S30 Fig — Each point summarises FST between mexicana ancestry tracts within a focal maize population and mexicana ancestry tracts within the local mexicana population sampled at the same site. Within-mexicana ancestry FST is presented separately for three subsets of the genome: introgression peaks found in the focal maize population only, peaks shared between the focal maize and at least 3 other maize populations, and a genomewide estimate. Notably, peaks where adaptive introgression is limited to the local population (‘1 population peaks’) do not have reduced FST to local mexicana haplotypes. (TIF) [file pgen.1009810.s037.tif]

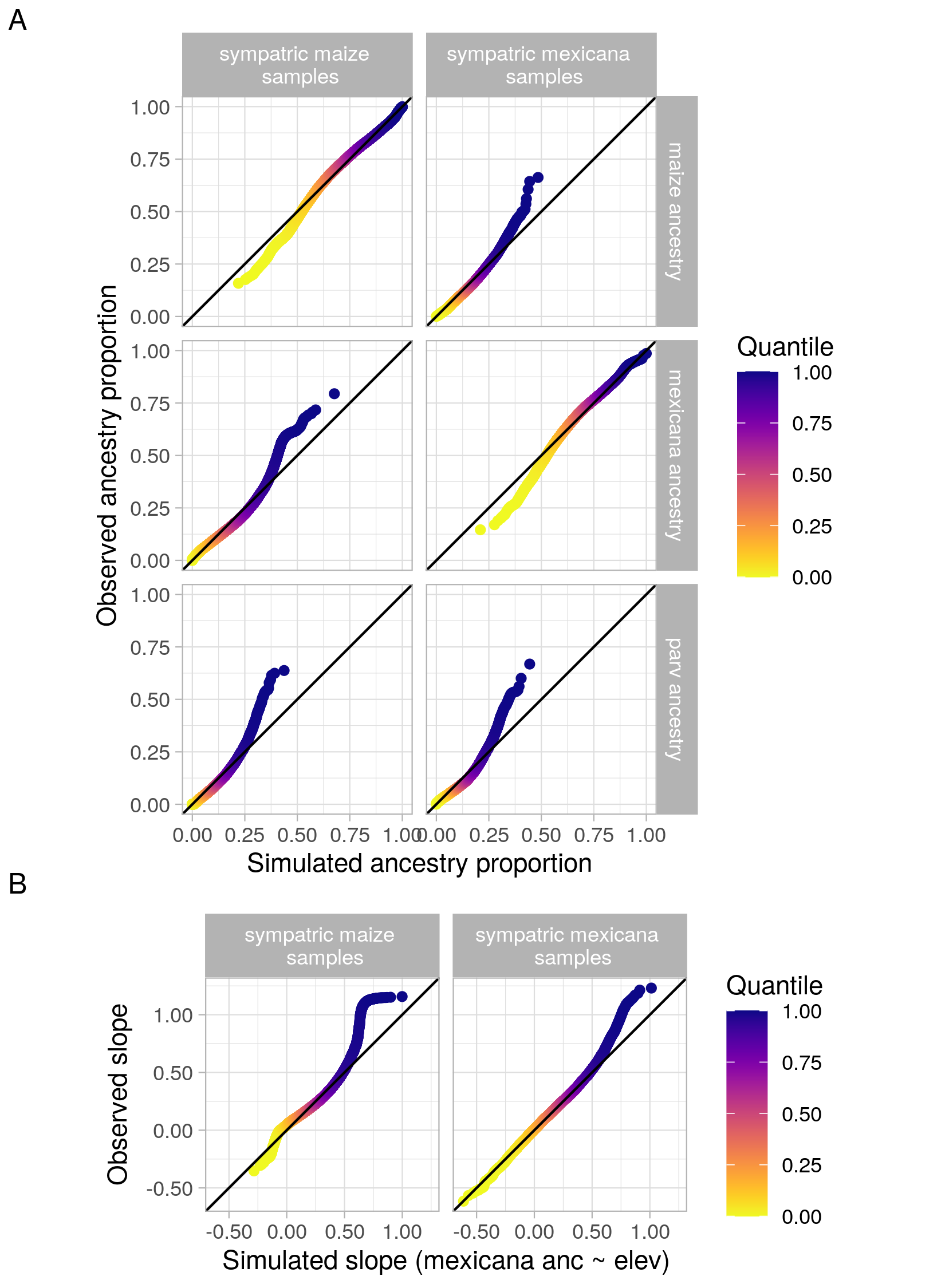

Supplement: S31 Fig — (A) QQ-plot of simulated vs. observed mean ancestry at individual loci across all sympatric individuals. (B) QQ-plot of simulated vs. observed slopes from the linear model mexicana ancestry ∼ elevation at individual loci. (TIF) [file pgen.1009810.s038.tif]

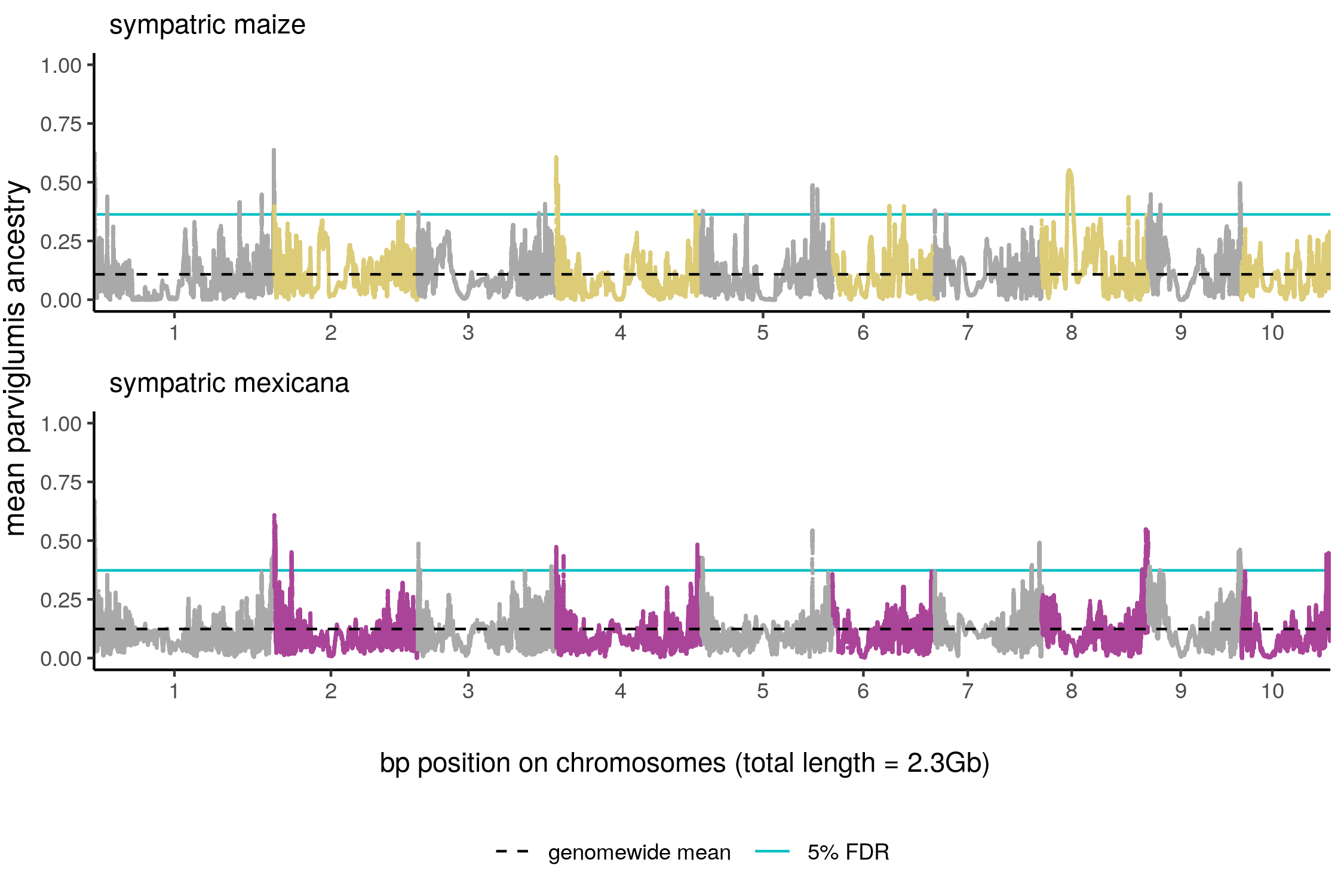

Supplement: S32 Fig — Mean introgressed parviglumis ancestry into sympatric maize and mexicana populations populations. The blue line shows the 5% false discovery rate for high introgression, set using multi-variate normal simulations. (TIF) [file pgen.1009810.s039.tif]

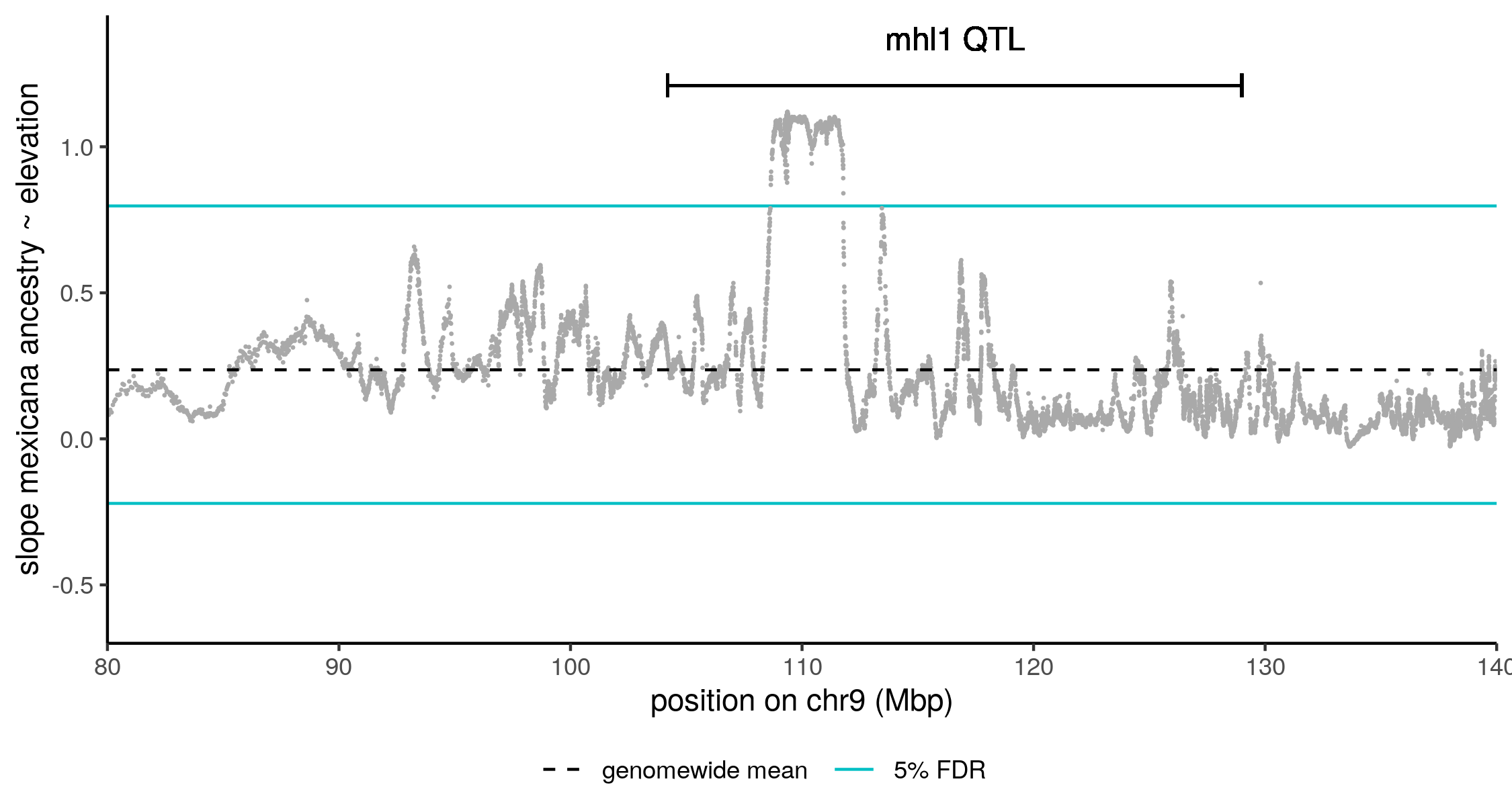

Supplement: S33 Fig — Slope of introgressed mexicana ancestry proportion in sympatric maize over a 1 km gain in elevation, zoomed in on the mhl1 QTL region on chromosome 9. Coordinates for the contiguous 3 Mb outlier region within this QTL are 9:108615836-111785557. (TIF) [file pgen.1009810.s040.tif]

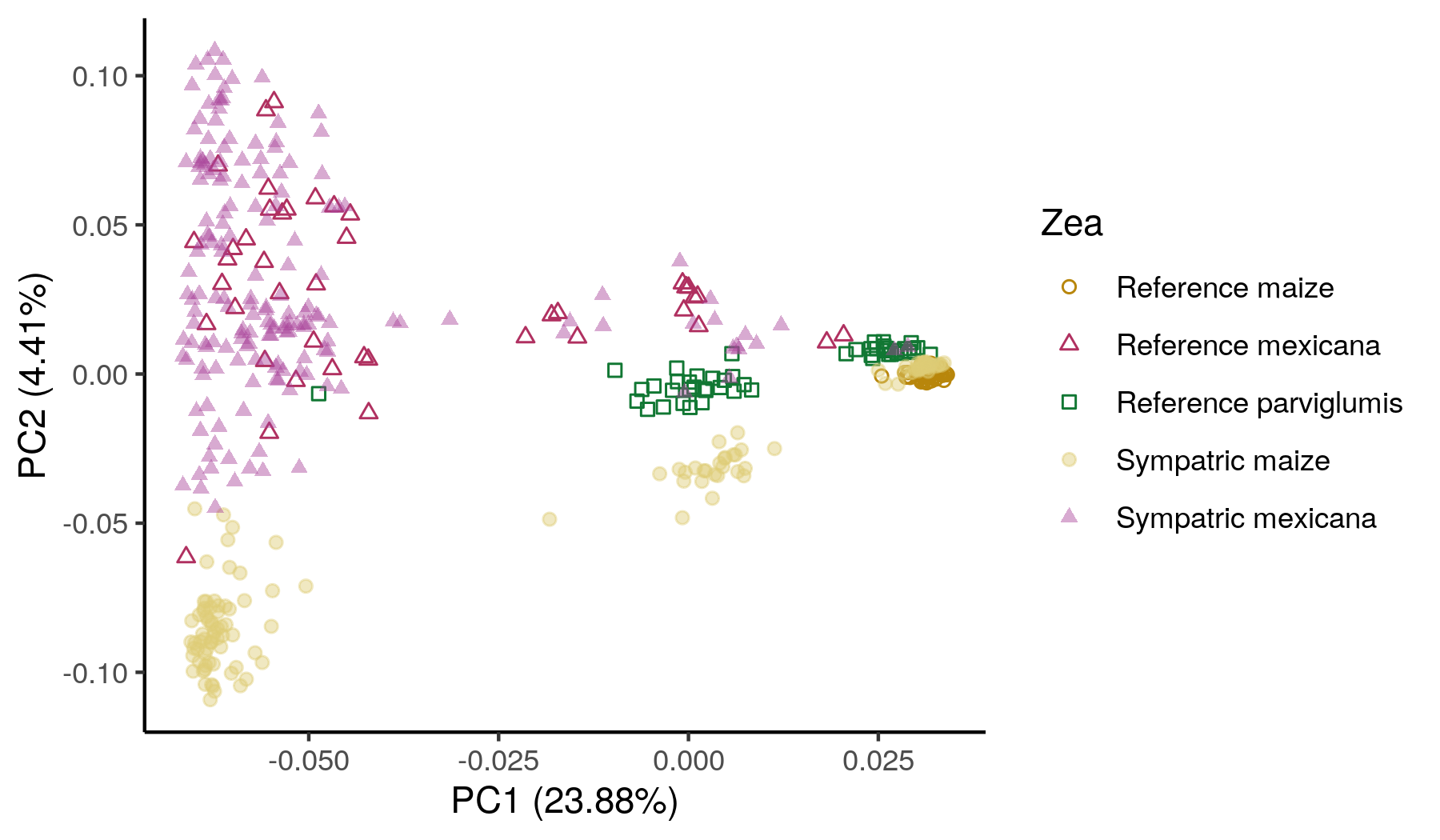

Supplement: S34 Fig — Principal components analysis of all SNPs in the 3 Mb outlier region within the mhl1 QTL region that shows a steep increase in introgressed mexicana ancestry across elevation (<5% FDR). This region on chromosome 9 is a putative inversion (9:108615836-111785557), separating out into three clusters across PC1: individuals homozygous for the common mexicana inversion allele (left), heterozygous individuals (middle) and individuals homozygous for the common maize inversion allele (right). There is evidence that the mexicana inversion allele segregates at low frequency in lowland parviglumis, but not lowland maize (right cluster includes all low-elevation reference maize). Additionally, PC2 primarily separates out diversity within the common mexicana allele for the inversion, with maize samples tending to have lower PC2 values. (TIF) [file pgen.1009810.s041.tif]

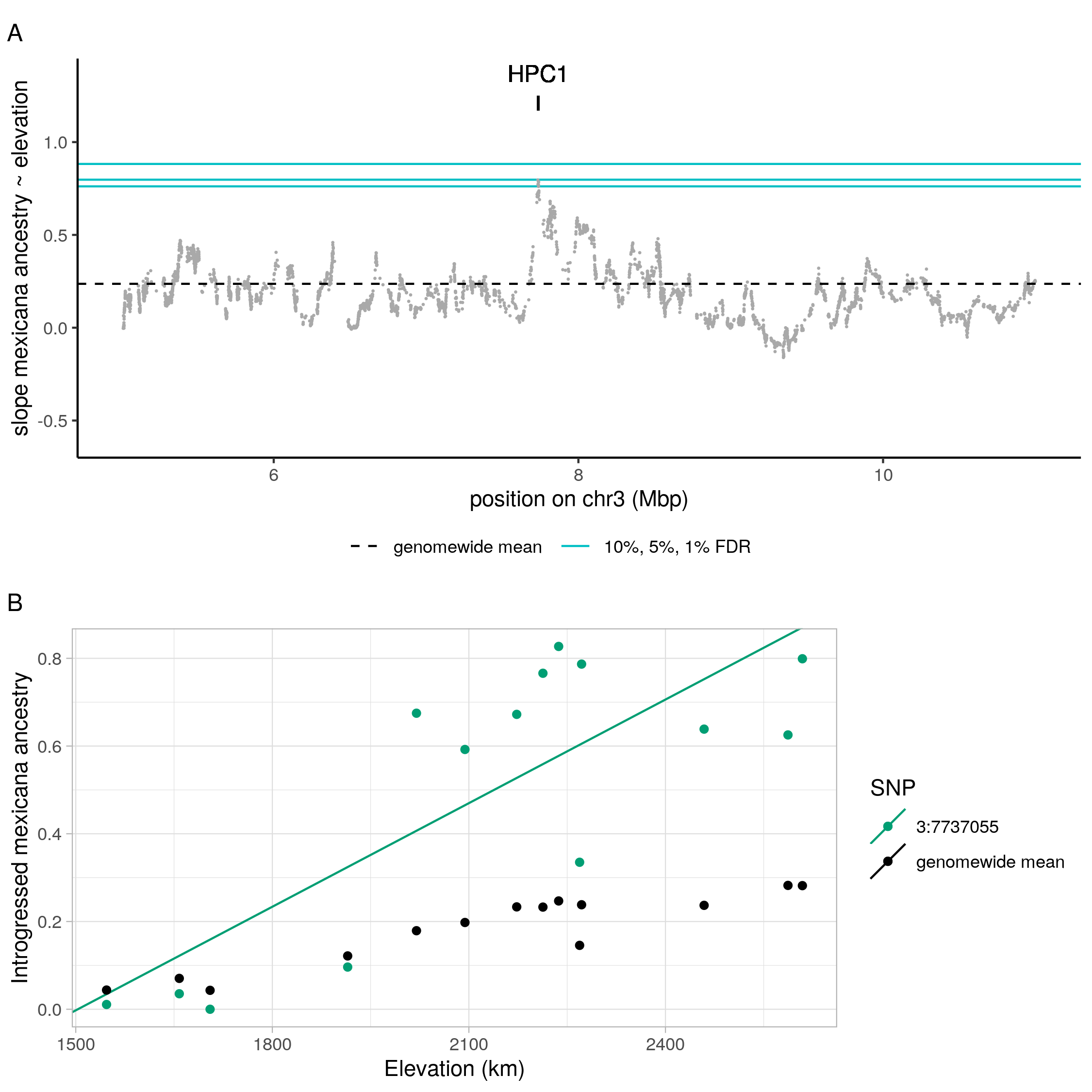

Supplement: S35 Fig — (A) Slope of mexicana ancestry across elevation near the HPC1 gene, with 1%, 5% and 10% FDRs for steep slopes indicated in blue. (B) Mean mexicana introgression proportions for each population, and fitted slope across elevation, for the top outlier SNP within HPC1 (3:7737055). In higher elevation maize populations, introgression at HPC1 far exceeds the genomewide mean mexicana ancestry (black circles). (TIF) [file pgen.1009810.s042.tif]

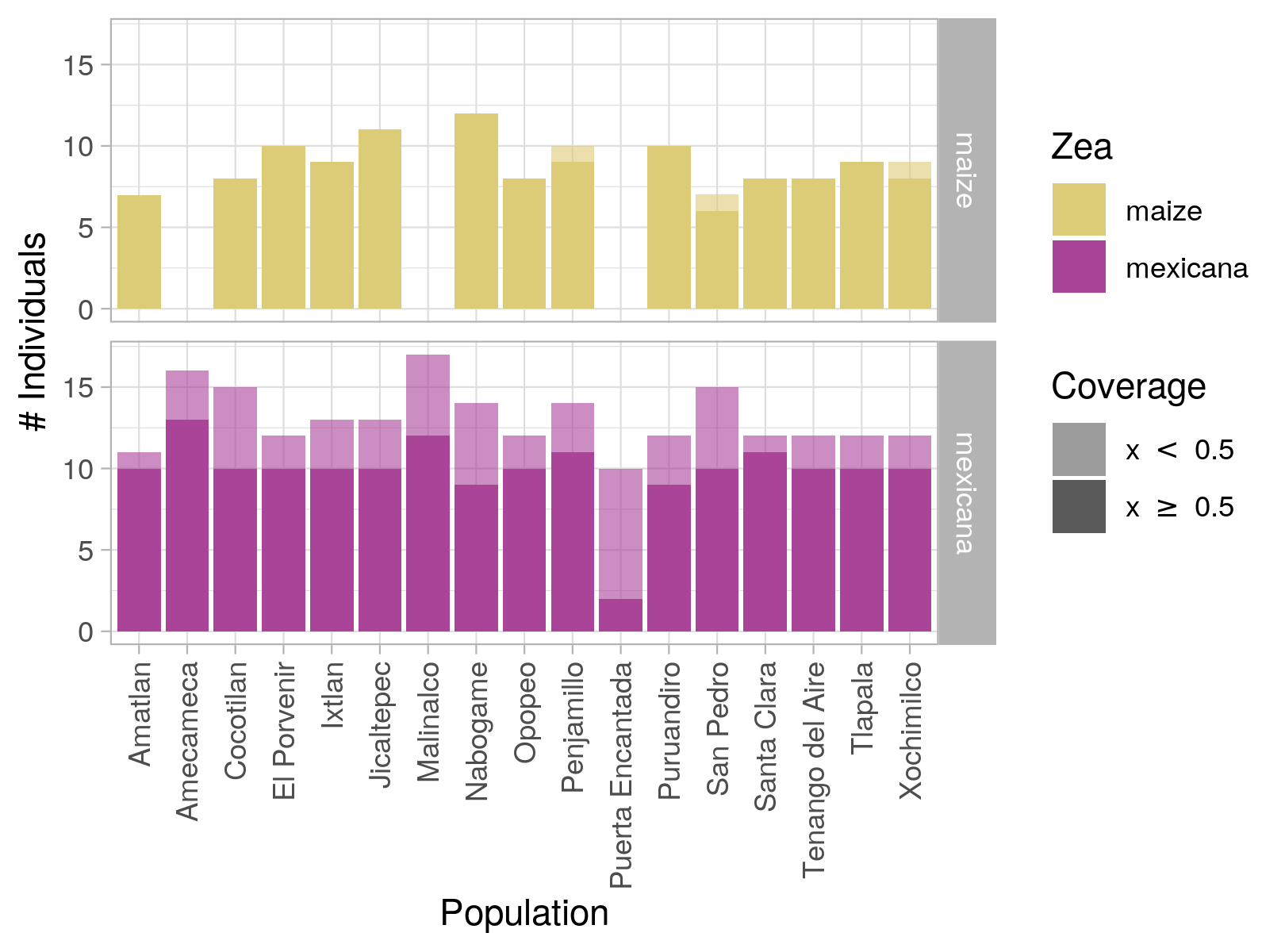

Supplement: S36 Fig — Number of maize (top) and mexicana (bottom) individuals sequenced by this study with minimum 0.05x WGS coverage. Amecameca, Malinalco and Puerta Encantada have no paired maize samples and are used as a reference panel for mexicana ancestry. For sympatric maize and mexicana, only individuals meeting a more stringent 0.5x coverage threshold (shown in darker shading) are included in analyses based on local ancestry inference. (TIF) [file pgen.1009810.s043.tif]

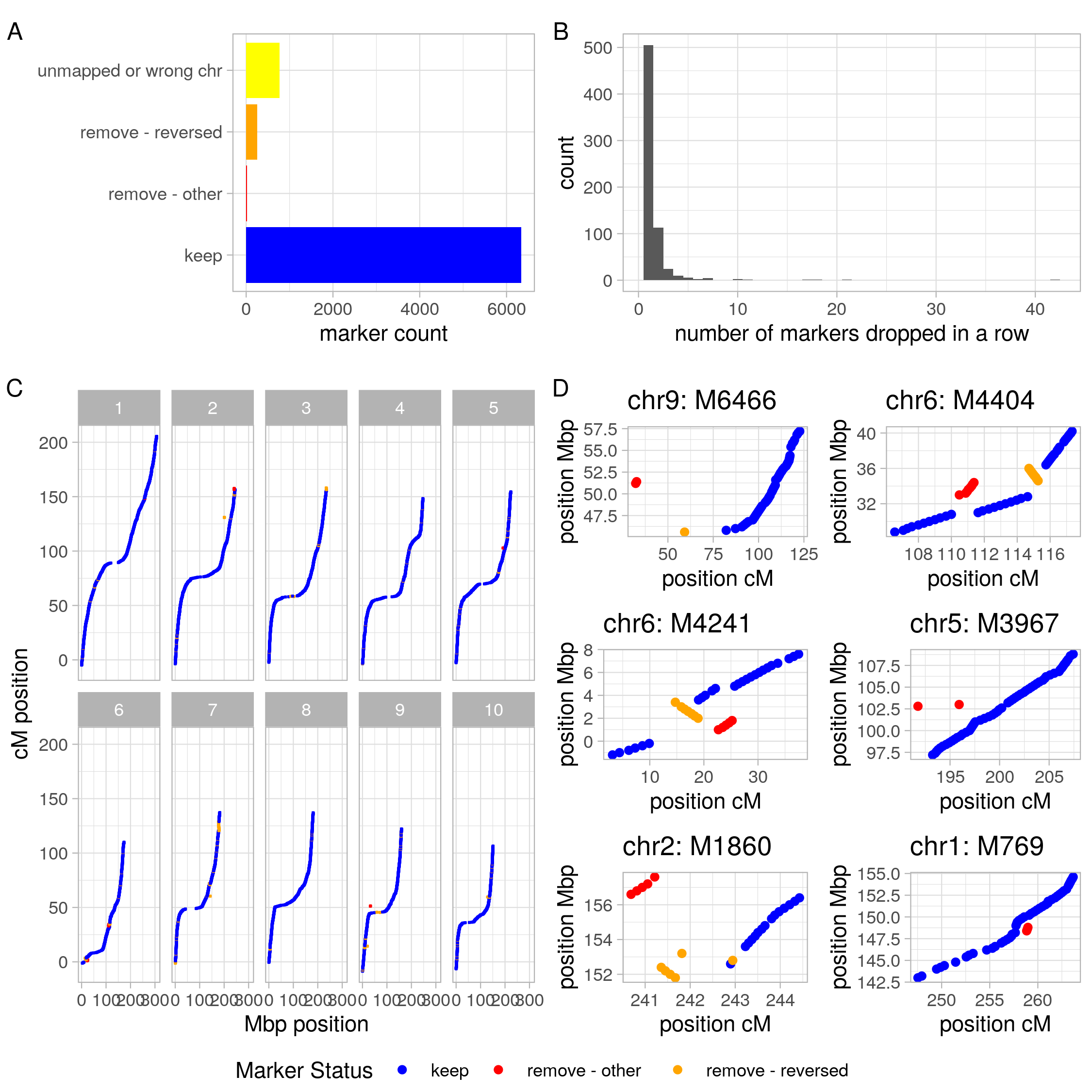

Supplement: S37 Fig — (A) Over 85% of the original markers from the Ogut et al [62] 0.2cM linkage map on reference genome v2 were successfully transferred to reference genome v4, using Assembly Converter (ensembl.gramene.org). We dropped markers automatically that mapped to the wrong chromosome or in reverse map order (presumably due to small contigs having corrected orientations in the newer version of the reference genome). A small number of markers were dropped by hand for more complex out-of-order mapping errors. (B) With few exceptions, the number of markers dropped in a row is small. (C) Visualization of the full linkage map on v4 of the reference genome. Included markers are in blue while excluded markers are highlighted in orange and red. (D) Zoomed in view of all 6 regions in the genome where out-of-order markers did not form simple reversals. For these more complex regions we identified markers to drop by hand (in red) to reach a monotonically increasing map solution. (TIF) [file pgen.1009810.s044.tif]

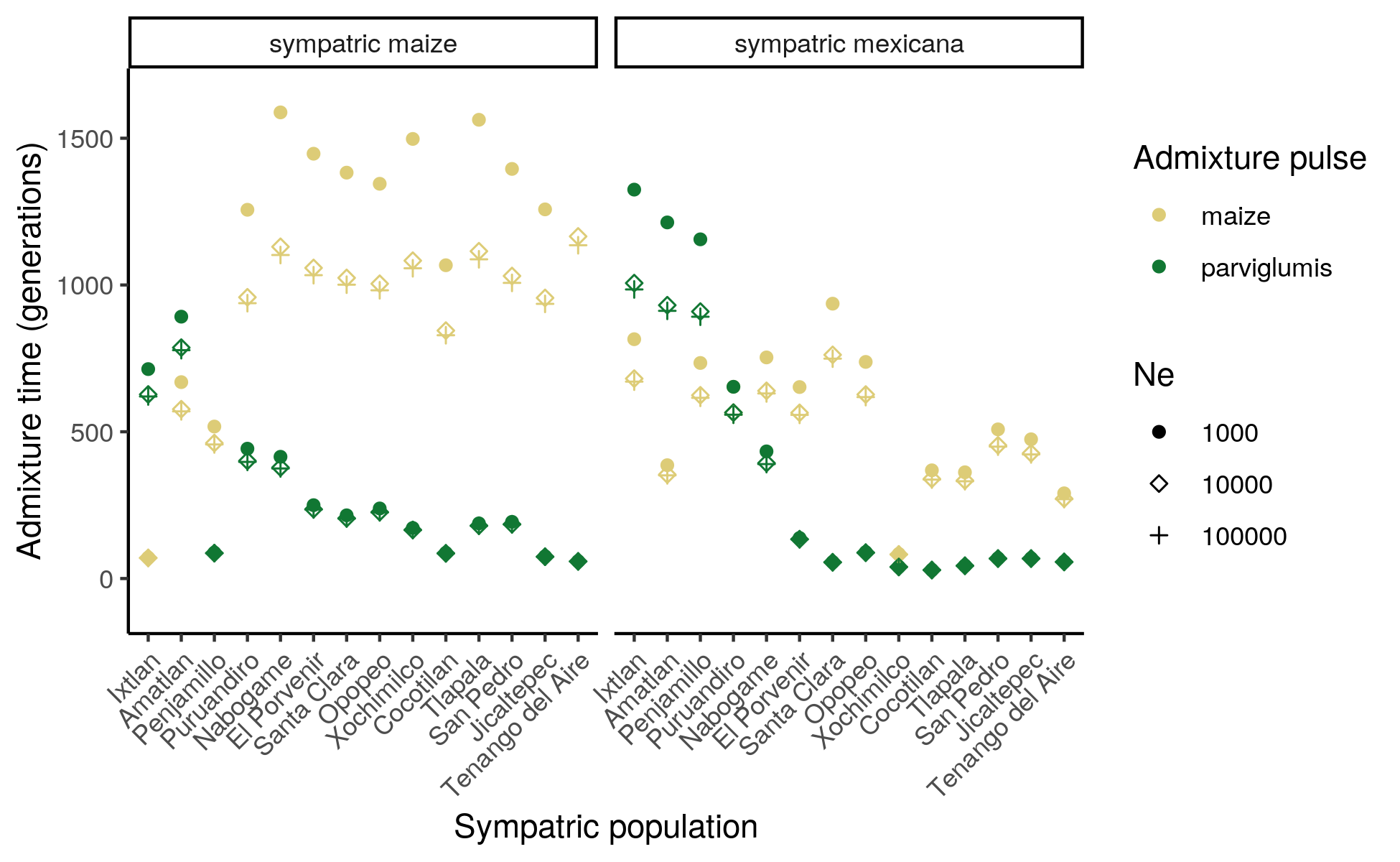

Supplement: S38 Fig — Admixture timing estimates from ancestry_hmm across 3 choices of effective population size. Timing estimates are strongly correlated with slightly older estimates for the smallest Ne = 1000. Local ancestry calls across the genome, summarised within sympatric maize and sympatric mexicana for each Ne, were all tightly correlated (r > 0.99). Main results of the paper are presented only for Ne = 10,000. (TIF) [file pgen.1009810.s045.tif]
